# Preparation, structural characteristics and immune regulatory effects of *Codonopsis pilosula* polysaccharides: a review

**DOI:** 10.3389/fimmu.2025.1641928

**Published:** 2025-09-22

**Authors:** Meitong Pan, Xiaozhuang Zhang, Shumin Huang, Meiqi Liu, Shiyi Song, Junbai Ma, Chenliang Li, Wei Ma, Xiubo Liu

**Affiliations:** ^1^ College of Pharmacy, Heilongjiang University of Chinese Medicine, Harbin, China; ^2^ College of Jiamusi, Heilongjiang University of Cahinese Medicine, Jiamusi, China

**Keywords:** Codonopsis pilosula polysaccharides, Polysaccharide preparation, Structural characteristics, Immunomodulation, Structural modification

## Abstract

*Codonopsis pilosula* polysaccharides (CPPs), a class of representative bioactive compounds derived from *Codonopsis pilosula*, have attracted considerable attention recently as natural immunomodulators due to their wide range of biological activities and favorable safety profile. This review provides a comprehensive overview of recent advances in the extraction and purification methods, chemical structural features, immunomodulatory mechanisms, and the impact of structural modifications on the immunological functions of CPPs. Notably, this work emphasizes the integration of structural modification strategies with immunomodulatory mechanisms, a perspective rarely highlighted in previous reviews. Special attention is given to the macrophage-centered TLR4/MyD88/NF-κB signaling pathway, which plays a pivotal role in coordinating adaptive immune responses through cytokine-mediated interactions with T and B lymphocytes. Structural modification strategies, such as sulfation, phosphorylation, selenization, and nano-carrier incorporation, have significantly enhanced the stability, bioavailability, and immunoregulatory effectiveness of CPPs. Furthermore, this review addresses current challenges, including structural heterogeneity, lack of standardization, and limited clinical evidence. This work aims to provide a valuable reference for future research and applications of CPPs as immunotherapeutic agents and functional food ingredients.

## 1 Introduction

*Codonopsis pilosula* (Franch.) Nannf., an herbaceous perennial vine belonging to the *Campanulaceae* family (1). *C. pilosula*,a traditional Chinese medicinal herb, has a long-history of use (2). The Pharmacopoeia of the People’s Republic of China describes C. pilosula as having a sweet taste and neutral properties, which influence the spleen and lung meridians (3). This herb has been traditionally utilized to enhance the function of the middle burner and increase qi, as well as to nourish the blood, produce body fluids, fortify the spleen, and hydrate the lungs (4). It is frequently used in clinical practice to address issues like spleen deficiency, fatigue, diminished appetite, breathlessness, and palpitations (5). With advances in modern medical research, the chemical constituents and pharmacological activities of *C. pilosula* have been increasingly elucidated (6).

Major bioactive components identified include polysaccharides, saponins, alkaloids, volatile oils, and various flavonoids (7). Among these, *C. pilosula* polysaccharides (CPPs) have recently gained particular attention as abundant, functionally significant, high-molecular-weight natural products (8). Interestingly, CPPs exhibit potent and broad immunomodulatory activities (9, 10). CPPs regulate the immune response via multiple targets and pathways, participating in the function of macrophages, T/B lymphocytes, and natural killer (NK) cells, and modulating the secretion of cytokines, activation of the complement system, and development of immune organs (15–18). Furthermore, CPPs may serve as a novel type of natural immunologic adjuvant, enhancing vaccine immunogenicity and showing great potential in disease prevention and immune modulation (19).

The bioactivities of polysaccharides bioactivities greatly depend on their molecular characteristics, including molecular weight, monosaccharide composition, glycosidic linkages, and the length and distribution of side chains. Hence, detailed structural characterization and analysis of the structure-activity relationships of CPPs are crucial for their precise applications (11, 12). Moreover, modern analytical techniques such as Fourier-transform infrared spectroscopy (FT-IR), nuclear magnetic resonance (NMR), and gas chromatography-mass spectrometry (GC-MS) have been developed, which not only aid in understanding the structure of CPPs but also lay the foundation for further studies on their immunological functions (13, 14).

In addition to structural studies, extraction and purification methods play a crucial role in determining the quality and bioactivity of CPPs. Hot water extraction (HWE) is a relatively simple and cost-effective method, but the extraction yield is relatively low, and the bioactivity is not well-preserved. With the development of new extraction methods, ultrasound-assisted extraction (UAE), microwave-assisted extraction (MAE), and enzyme-extracted aqueous solution (EAE) have been developed (15–18). These methods offer more efficient alternatives to traditional extraction techniques. Additionally, techniques like column chromatography, ion-exchange chromatography (IEC), and gel filtration chromatography (GFC) have been employed to purify CPPs, enabling high-quality and stable preparations suitable for downstream structural and functional studies (19, 20).

Recent advancements in CPP research extend beyond natural extraction methods to include structural modifications (21). Chemical derivatization approaches, including sulfation, phosphorylation, and selenization, are employed to enhance the immunological activity, bioavailability, and stability of CPPs. Furthermore, nanotechnology, such as CPP-loaded nanoparticles or polysaccharide-based nanocomposites, offers novel strategies for targeted delivery systems and immunopotentiation (22–24). These abundant modification methods provide a theoretical basis and technical reference for further research on CPPs in pharmaceuticals, functional foods and immunotherapy (25).

In conclusion, CPPs are naturally occurring, immunologically active substances that are widely sourced, have high safety, low toxicity, and possess a wide range of biological activities. They hold substantial research value and promising application prospects. This article aims to summarize recent advances in the extraction, structural characterization, immunological activities, and structural modifications of CPPs, providing a theoretical and technical basis for further research on their functional development and immunomodulatory applications.

## 2 Extraction

**Figure 1:**
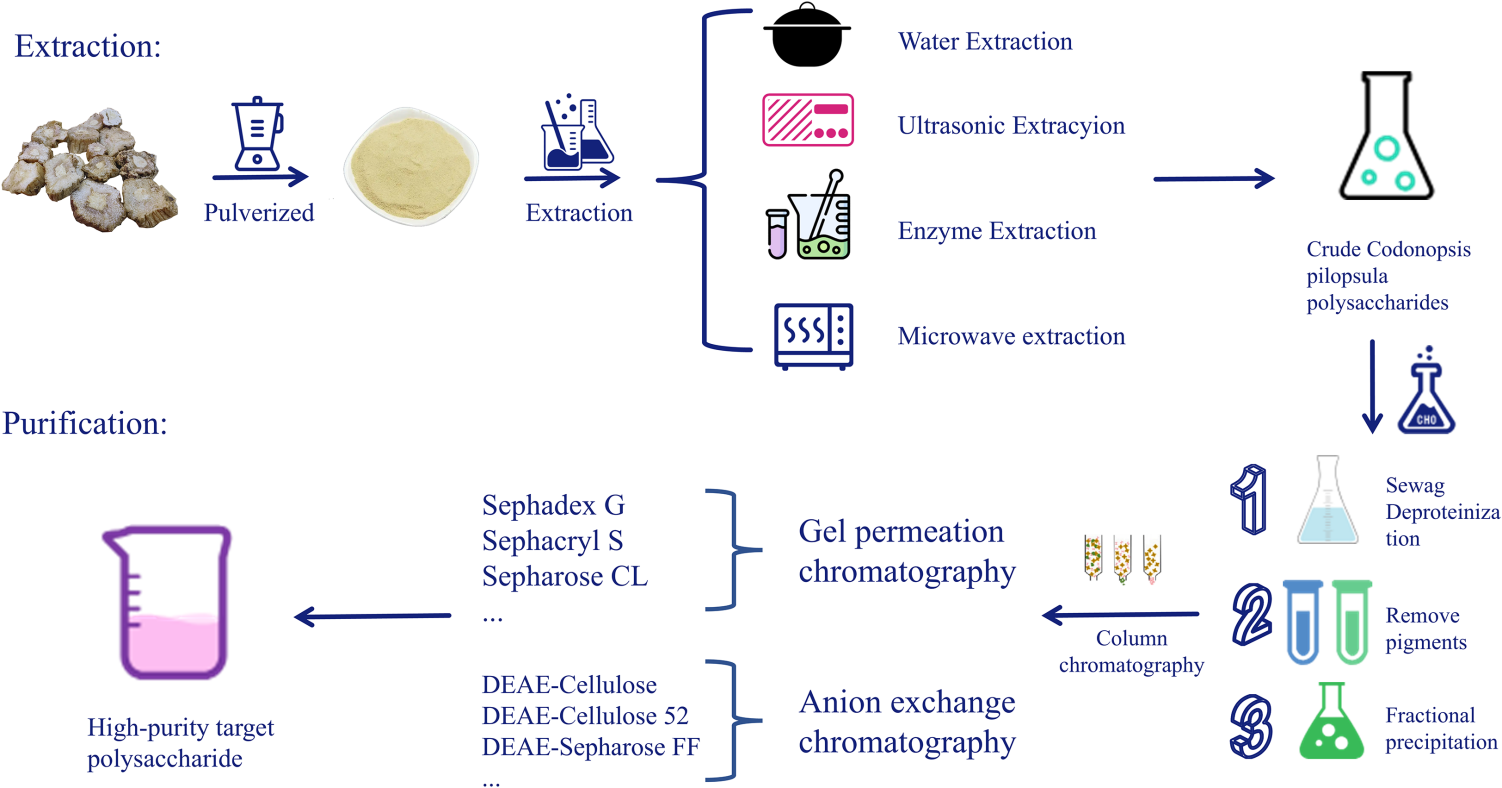
Preparation process of CPPs.

**Figure 2:**
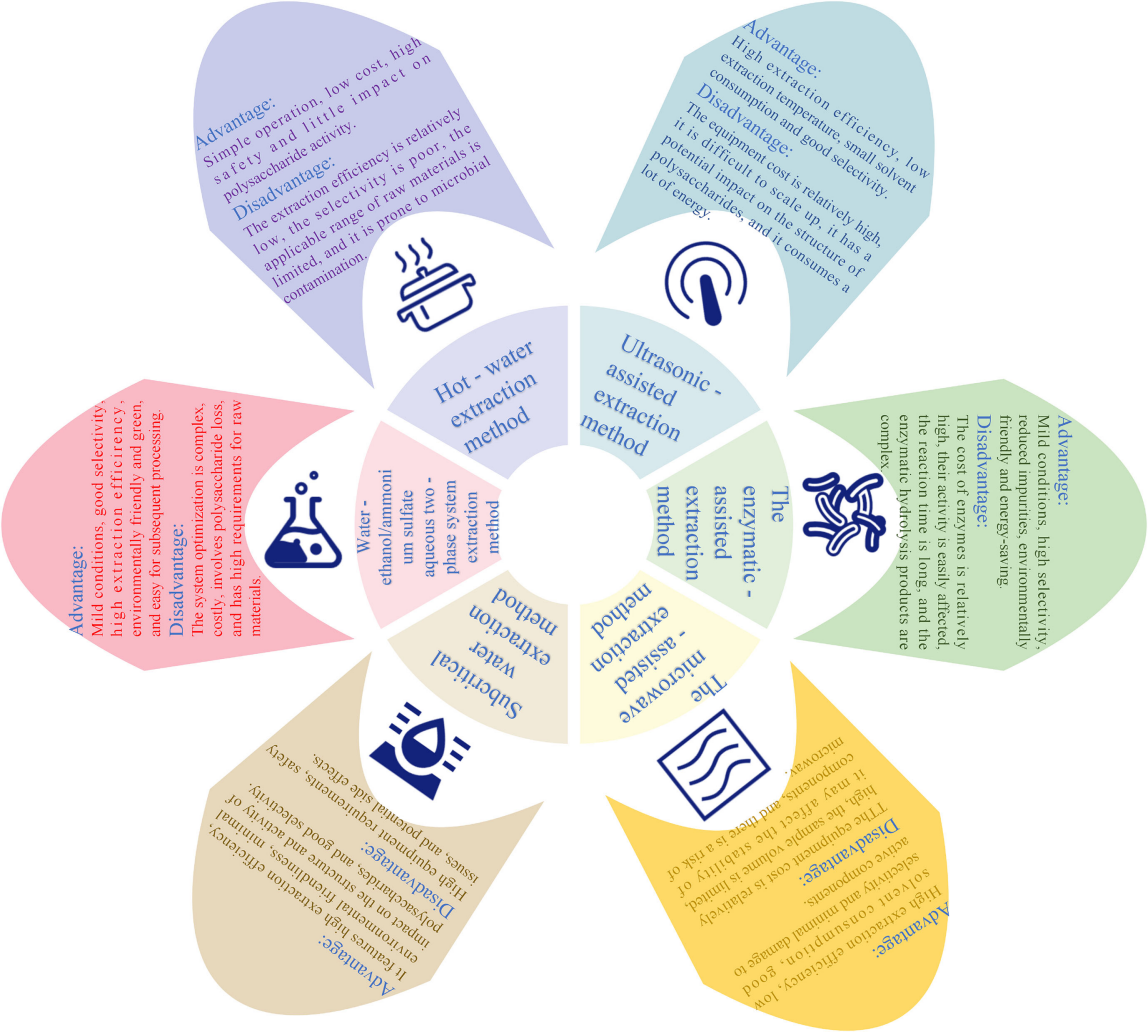
Advantages and disadvantages of different extraction methods for CPPs.

Polysaccharides are high-molecular-weight substances composed of multiple monosaccharide molecules connected by glycosidic bonds and widely exist in nature (26). HWE, EAE, UAE, and MAE are the general procedures adopted for polysaccharide extraction (**Figure 1**) (27–29). Every method possesses special characteristics and advantages, and the selection of an appropriate extraction procedure is thus crucial (**Figure 2**) (30). To better optimize extraction efficiency and performance, scientists often apply statistical optimization methods, such as orthogonal experimental design (OED) and response surface methodology (RSM) (8). These techniques can optimize extraction conditions to promote yield and bioactivity, thereby meeting various demands of research and industrial applications (31).

### 2.1 Hot water extraction

HWE is the most widely used method for CPP extraction (32, 33). It is simple to operate, inexpensive, and highly efficient and is suitable for the extraction of most plant polysaccharides (15). Optimization of extraction conditions is necessary for improving the extraction efficiency and quality of polysaccharides (34). For instance, the ideal rise in extraction temperature enhances the solubility of polysaccharides; however, excessive temperatures trigger degradation. Similarly, extended extraction time guarantees the total release of polysaccharides, while excessive exposure will decline bioactivity. Adjusting the solid-to-liquid ratio improves the efficiency of solubility and reduces solvent wastage, while repeated cycle extraction enhances yield (35). Experiments revealed that, under optimized conditions of 80 °C, a solid-to-liquid ratio of 1:16, an extraction time of 80 minutes, and a particle size of 180 μm, the maximum yield of CPPs reached 15.56% (36). Shi et al. optimized RSM extraction conditions and found optimum parameters of extraction time 2.3 h, extraction temperature 89 °C, and solid-to-liquid ratio 1:24, under which 15.66% CPPs existed (37). Similarly, Fu et al. employed RSM to optimize fructan extraction from *C. pilosula* and reported a yield of 20.6% obtained with an extraction temperature of 100 °C, a solvent-to-material ratio of 40 mL/g, and an extraction time of 2.5 hours in two cycles (38). Li et al. further optimized extraction conditions and the authors concluded that an extraction temperature of 85 °C, an extraction time of 1.5 hours, and a solid-to-liquid ratio of 1:12 provided a final yield of 22.57% after two extraction cycles (39). Another work reported achieving a 25.7% final yield at 90 °C, using an extraction time of 45 minutes and a solid-to-liquid ratio of 1:20 (40). In conclusion, the optimization of extraction parameters, such as temperature, time, solid-to-liquid ratio, and extraction cycles, useing OED and RSM is a primary focus in maximizing of both the efficiency and quality of CPPs extraction.

### 2.2 Ultrasound-assisted extraction

The UAE utilizes the cavitation process and the mechanical force of ultrasound waves to rupture the cell walls of plant cells, improve the penetration of the solvent, and permit the release of polysaccharides (41). Compared to conventional extraction technologies, UAE offers numerous advantages including higher extraction rates, lower energy utilization, and improved yield, making it easier to operate and more environmentally friendly (42). In addition, UAE lowers the degradation of heat-sensitive polysaccharides and preserves their biological activity, making it an extremely efficient and sustainable extraction technique (43). Cao et al. investigated the effects of various factors, such as the solid-to-liquid ratio, ultrasonic power, extraction time, and temperature, on the yield of CPPs in single-factor experiments. They further optimized the extraction conditions and found the best parameters to be:solid-to-liquid ratio = 1:30 (g/mL), power of ultrasonication = 280 W, time of extraction = 75 minutes, and temperature of extraction = 74 °C. Under the above conditions, the extraction yield was 18.42% (16). Similarly, Wang et al. also performed single-factor tests to investigate the influence of the temperature, the solid-to-liquid ratio, extraction time, and ultrasonic power on extraction efficiency. They then optimized the extraction process using a uniform design method, and found the optimum conditions to be a 70 °C temperature, a 25:1 solid-to-liquid ratio, 35 minutes of extraction time, and 135 W of ultrasonic power. These conditions produced an average extraction yield of 31.26% (44). Furthermore, Wang et al. demonstrated that UAE with an ultrasonic power of 180 W, a frequency of 60 kHz, and a treatment time of 30 minutes increased the content of CPPs by 14.7%, while also effectively preserving polysaccharide bioactivity and other functional ingredients (45). In conclusion, UAE is an extremely effective, environmentally friendly, and green extraction technique that holds a vast potential for the extraction of CPPs. Further research and optimization of UAE parameters can contribute to enhancing the functional use and industrial applications of CPPs.

### 2.3 Enzyme-assisted extraction

EAE utilizes specific enzymes for the degradation of plant cell walls or other structural components, thereby facilitating the efficient release of target molecules under mild conditions (46). This method has several advantages, including high extraction efficiency, high selectivity, and the ability to retain heat-sensitive bioactive compounds,making it widely applicable for polysaccharide extraction (47, 48). Fan et al. optimized the polysaccharide extraction process from yeast-fermented fresh *C. pilosula* through single-factor experiments combined with RSM. The results indicated that the optimal extraction condition was 63 °C of temperature, 6 hours of extraction time, and 4% of enzyme concentration. Under these conditions, extraction yield was as high as 17.46%, significantly higher than the 12.69% achieved by conventional HWE (49). Gao et al. also optimized the extraction of CPPs by single-factor experiments combined with RSM. The optimal conditions were found to be an enzyme concentration of 0.2%, an enzymatic hydrolysis time of 1.5 hours, a hydrolysis temperature of 50 °C, and a pH of 4.2. Under the optimal conditions, the extraction yield was 25.23% (18). In general, compared to traditional extraction methods, EAE is energy-efficient and environmentally friendly, lowering impurities and reducing extraction time. Besides, the simplicity of enzyme selection based on raw material characteristics will enable better extraction efficiency and quality of polysaccharides. Continued advances in enzyme selection and process optimization will also contribute to the broader application of CPPs in various applications.

### 2.4 Other extraction methods

Apart from the already described extraction methods, scientists arecontinually seeking simpler and faster ways to obtain CPPs. For instance, MAE induces fast oscillation of polar molecules inside the material using microwave electromagnetic fields, therefore producing both thermal and non-thermal effects. The rapid heating of the solvent, material causes cell wall rupture and thereby increasing extraction efficiency (50, 51). Yu et al. examined CPPs extraction by OED about solid-to-liquid ratio, microwave radiation power, and extraction time. Water was found to be the ideal solvent; a temperature of 70 °C, a solid-to-liquid ratio of 1:40, an extraction period of 20 minutes, and a microwave power of 500 W, therefore producing an extraction rate of 14.8% (17). Utilizing the lowered polarity of water at high temperatures and pressures, subcritical water extraction (SWE) extracts bioactive compounds. Particularly appropriate for heat-sensitive materials, this approach is also environmentally benign and reasonably affordable (52, 53). By employing a temperature of 150 °C, an extraction duration of 45 minutes, and a liquid-to-material ratio of 12 mL/g, Zhang et al. refined the SWE process for CPPs, thereby establishing optimal conditions and an extraction rate of 19.51% (54). A liquid-liquid extraction system that generates immiscible phases between ethanol and ammonium sulfate, therefore allowing the separation of target chemicals, is the water-ethanol/ammonium sulfate aqueous two-phase system (55, 56). Using this approach, Lu et al. extracted CPPs by optimizing the conditions to a concentration of 17% ammonium sulfate, 30% ethanol, an extraction temperature of 40 °C, and a pH of 6. Under these circumstances, the extractive yield was 31.57% (57). In conclusion, these advanced extraction methods offer higher efficiency and selectivity compared to traditional techniques, enabling the extraction of bioactive compounds under milder conditions. These techniques show great promise for both commercial and medicinal uses, as they enhance the bioactivity of polysaccharides and increase extraction efficiency.

## 3 Separation and purification

The crude polysaccharides thus obtained through the above-discussed methods may also contain contaminants such as proteins, pigments, monosaccharides, inorganic salts, and lipids (56). These impurities may interfere with the structural analysis and bioactivity tests of the polysaccharides;therefore, adequate separation and purification are essential (58). General purification methods involve the extraction of polysaccharides with 80%–95% ethanol for several hours to remove oligosaccharides and low-molecular-weight constituents (59). Protein impurities can be removed by the Sevag procedure or trichloroacetic acid precipitation, and pigments can be eliminated by hydrogen peroxide oxidation or adsorption to a macroporous resin (60). Further purification may be achieved by precipitation techniques or column chromatography (31). EPM is a standard procedure for fractionating crude polysaccharides, whereby polysaccharides with different molecular weights precipitate successively as the concentration of the organic solvent increases (61). Column chromatography techniques, including IEC and GFC, are employed frequently (28). IEC isolates acidic and neutral polysaccharides using gradient elution using sodium chloride solutions, and GFC fractionates polysaccharides based on molecular weight differences (19, 20, 62).

Briefly, the extraction of CPPs typically involves several key steps. Initially, *C. pilosula* is thoroughly washed and dried at 40 °C in an oven or vacuum dryer, then powdered. The powder is defatted twice with 95% ethanol successively for two hours each to remove pigments, oligosaccharides, lipids, and other small-molecule compounds. The resulting residue is then subjected to three cycles of HWE (each lasting two hours), filtered, and concentrated. The crude polysaccharides are ethanol-precipitated, recovered by centrifugation, and purified by removing protein with the Sevag method and activated carbon adsorption for decolorization. Dialysis, evaporation, ethanol precipitation, filtration, and freeze-drying operations yield crude CPPs. The crude CPPs, for further purification, are dissolved in double-distilled water and subjected to IEC (e.g., DEAE-cellulose, DEAE-Sepharose) and GFC (e.g., Sephadex G, Sephacryl S). The purified polysaccharides are subsequently harvested by buffer elution, dialyzed, concentrated, and freeze-dried to obtain high-purity CPPs (8, 63, 64).

## 4 The structural characteristics of CPPs

Polysaccharides are complex carbohydrates composed of monosaccharide residues linked via glycosidic bonds. Key structural characteristics include molecular weight, monosaccharide composition, glycosidic linkage types, and their specific arrangement. In recent years, various analytical methods have been utilized to elucidate the chemical architecture of CPPs.

### 4.1 Molecular weight

The molecular weight of polysaccharides plays a crucial role in determining their biological activities and is therefore considered an important parameter reflecting their physicochemical properties and functional potential (65). Determining molecular weight facilitates a deeper understanding of the relationship between polysaccharide structure and function and is commonly estimated using statistical averaging methods (60). Traditional techniques, such as viscometry, light scattering, and osmometry, have been widely used, however, they tend to be complex and susceptible to experimental errors  (66, 67). Currently, more accurate and reliable methods are preferred, including high-performance liquid chromatography (HPLC), gel permeation chromatography (GPC), high-performance gel permeation chromatography, and high-performance size-exclusion chromatography (14, 38, 68, 69). Due to variations in the types of CPPs, as well as differences in extraction, purification processes, and analytical methods, the reported average molecular weights of CPPs vary across studies (12, 70). For instance, in hot-water extraction methods, temperature and extraction duration significantly affect the resulting molecular weight. Increasing the temperature and extending the extraction time within an appropriate range can enhance the yield of high-molecular-weight polysaccharides. However, excessive heat may cause polysaccharide degradation, resulting in a decrease in molecular weight (71). Zou et al., for example, extracted pectic polysaccharides from *C. pilosula* roots at 50 °C and 100 °C, yielding 50WCP-II-I (71.6 kDa) and 100WCP-II-I (53.2 kDa), respectively, highlighting the influence of temperature on molecular size. Moreover, the pore size of ultrafiltration membranes can also affect the molecular weight distribution of polysaccharides (72). Li et al. separated CPPs into three fractions using membranes with different molecular weight cut-offs, yielding CPPS-I (< 60 kDa), CPPS-II (60–100 kDa), and CPPS-III (> 100 kDa), demonstrating the impact of membrane filtration on polysaccharide size profiling (14).

### 4.2 Monosaccharide composition

Monosaccharide composition serves as a fundamental basis for elucidating the structural features, physicochemical properties, and structure-activity relationships of polysaccharides (73). Variations in purification techniques or raw material sources can lead to differences in monosaccharide ratios, glycosidic bond types, and linkage sequences (28). To analyze the monosaccharide profile of CPPs, commonly employed techniques include gas chromatography (GC), HPLC, NMR, and IR (74, 75). Su et al. separated a homogeneous polysaccharide, CPP-1, from the roots of *C. pilosula*, which consisted of mannose (Man), glucose (Glc), galacturonic (Gal), and arabinose (Ara) with a molar ratio of 5.86:51.69:34.34:8.08 (70). Similarly, Bai et al. isolated two polysaccharides, CPP1a and CPP1c; CPP1a consisted of rhamnose (Rha), Ara, galacturonic acid (GalA), Gal, and Glc in a molar ratio of 1:12:1:10:3. However, CPP1c had a higher proportion of GalA, which may be accountable for its more active biological activity compared to CPP1a (11). Zhang et al.identified eight monosaccharides present in CPPs, namely Man, Rib, Rha, glucuronic acid, GalA, Glc, Gal, and Ara,with Glc being the most prevalent at 49.87%. The molar ratio of the monosaccharides was found to be 0.53:0.60:0.51:3.50:9.55:21.73:4.78:2.38 (74).

### 4.3 Structural characteristics

**Figure 3:**
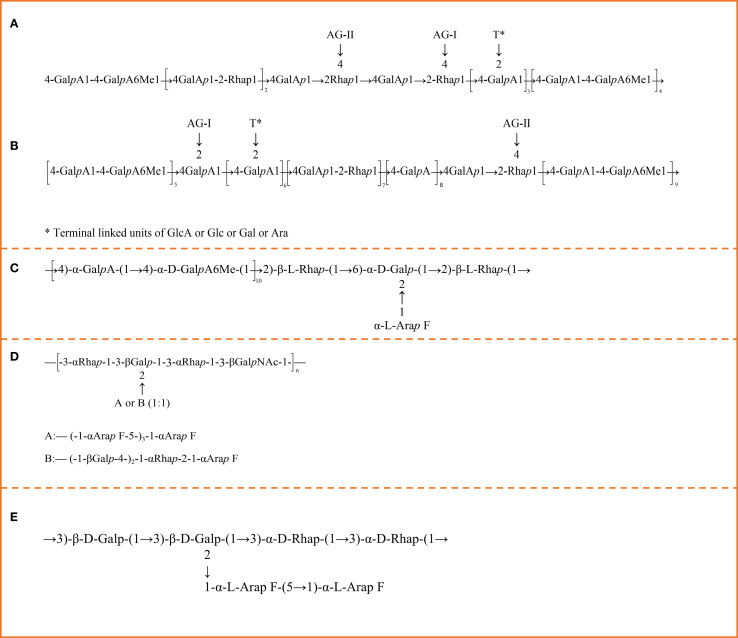
**(A)** Predicted structure of 50WCP **(B)** Predicted structure of 100WCP **(C)** Predicted structure of CPP1b **(D)** Predicted structure of CPPS3 **(E)** Predicted structure of CPP.

Due to the complex branching and linkage patterns of different CPPs, structural characterization is typically challenging (8). Structural analysis of polysaccharides primarily involves the identification of monosaccharide linkage sequences and glycosidic bond conformations. Common analytical techniques for CPPs structural elucidation include FT-IR, NMR, and GC-MS (9, 11, 70). A full structural characterization of polysaccharides typically requires the integration of multiple analytical techniques. FT-IR spectroscopy provides valuable information regarding functional groups and bond types within the polysaccharide molecule (76). The broad band of absorption at 3300–3450 cm^−^¹ corresponds to the stretching vibration of the free hydroxyl (-OH) group, indicative of the high hydroxyl content in polysaccharides, which is directly related to their solubility in water (77). Bands of absorption 2800–2950 cm^−^¹ are attributed to C-H stretching vibrations (methyl or methylene groups), which correspond to the carbon skeleton of the sugar ring (76). Common signals in 1000–1200 cm^−^¹ correspond to the pyranose ring and glycosidic linkage stretching vibrations (77, 78), while 1025–1152 cm^−^¹ bands of absorption also confirm the presence of pyranose rings (38, 70). In addition, bands at 852 cm^−^¹ and 893 cm^−^¹ of absorption are characteristic of the presence of α- and β-glycosidic linkages, respectively (14, 76). Bands at 1600–1750 cm^−^¹ show vibrational characteristics of carboxyl (C=O) and ester (COOCH_3_) groups, which are usually present in pectic polysaccharides (12, 79, 80). Detailed determination of glycosidic bond configuration and linkage structures is achieved with ¹H NMR spectroscopy (81). The chemical shift of the anomeric carbon (C1) can determine the configuration of the sugar ring, and α-anomers would typically be in the range of 5.0–5.5 ppm (14), whereas β-anomers appear in 4.5–5.0 ppm (76). ¹H NMR also identifies signals for branched structures or distinct functional groups such as methylated high-galacturonic regions (11). GC-MS also aids in polysaccharide characterization, providing quantitative information on monosaccharide composition. The interplay of these analysis techniques gives a clearer vision of the structural complexity of polysaccharides (82, 83). Zou et al. isolated two pectic polysaccharides, 50WCP-II-I and 100WCP-II-I, from the roots of *C. pilosula*. The backbone of 50WCP-II-I primarily consists of a long homogalacturonan (HG) region composed of GalA units, some of which are methyl-esterified. Embedded within the HG backbone are short rhamnogalacturonan-I (RG-I) regions, in which Rha residues at the O-4 position are substituted with arabinogalactan type I (AG-I) and arabinogalactan type II (AG-II) side chains (**Figure 3A**). In contrast, 100WCP-II-I contains two major branched regions: one in which GalA residues at the O-2 position are substituted with AG-I side chains and another where Rha residues at the O-4 position are substituted with AG-II side chains in the RG-I domain. The backbone also contains methyl-esterified HG region, and Rha residues are mainly 1,2-linked (**Figure 3B**) (72). Yang et al. Found another pectic polysaccharide, CPP1b, from *C. pilosula*, whose backbone is constituted by 1,4-linked α-D-GalA and methyl-esterified α-D-GalA, alternately interspersed with minor amounts of 1,2-linked β-L-rhamnose, 1,2,6-linked α-D-Gal and terminal α-L-Ara. Methylation analysis and NMR spectroscopy results demonstrated that side chains contain Ara and branched Gal residues (**Figure 3C**). In summary, the overall structure can be considered as a pectin containing RG-I-type rhamnogalacturonan regions consisting of a linear backbone with sparse branching. Ultrastructurally, CPP1b were amorphous, ovoid granules with sheet-like surface morphology (83). CPPS3, a water-soluble polysaccharide extracted from the roots of *C. pilosula*, was isolated. Its branched backbone is constituted by (1→3)-linked β-N-acetylgalactosamine, (1→3)-linked α-rhamnose and (1→2,3)-linked β-Gal. The degree of branching of this water-soluble polysaccharide is approximately 26%, meaning that one side chain exists for every four backbone residues. Its side chains consist of (1→5)-linked α-arabinofuran, (1→4)-linked β-Gal and (1→2)-linked α-rhamnose–Ara disaccharide chains. Its side chains were found to contain β-glycosidic linkages by infrared spectroscopy (**Figure 3D**) (84). Sun et al. Found a water-soluble polysaccharide from *C. pilosula* roots. Its backbone is constituted by (1→3)-linked β-D-pyranogalactose, (1→2,3)-linked β-D-pyranogalactose and (1→3)-linked α-D-pyranorhamnose. The O-2 position of Gal residues is substituted, in a 1:1 ratio, with side chains of a disaccharide, which consists of (1→5)-linked α-L-arabinofuranose units (**Figure 3E**) (85).

## 5 Immunological activity

Immunomodulation is the most striking biological activity of CPPs. So far, it has been reported that CPPs can significantly modulate the immune system through multiple pathways and mechanisms, which involve various components of the immune system, including immune cells, immune organs, immune factors and complement. Although CPPs exhibit multidimensional immune-regulatory effects, evidence indicates that their primary immunomodulatory role centers on macrophage activation, which in turn initiates and orchestrates subsequent adaptive immune responses through cytokine secretion and antigen presentation (**Table 1**).

**Table 1:** Summary of the immunomodulatory activity of CPPs.

Name	Model	Dosage	Effect	Rif.
CPPs-D1N1	Cyclophosphamide-immunosuppressed mice	200 mg/kg (oral)	Modulated serum immunoglobulins and cytokines; improved gut microbiota.	(9)
RCNP, RCAP-1/2	RAW264.7 macrophages	1.6–1000 μg/mL (*in vitro*)	RCAP-1/2 induced NO production; RCNP inactive.	(12)
WCP-I/Ia	C3H/HeJ mice	100 mg/kg (oral); Peyer’s patch cells (*in vitro*)	Elevated IL-6, TGF-β, TNF-α; increased spleen index and sIgA.	(80)
CPPS-I/II/III	B16F10 tumor-bearing mice	CPPS-II:10 mg/kg (i.p.); DOX:1 mg/kg (i.v.)	Enhanced macrophage polarization; suppressed tumor growth.	(14)
CPP	BALB/c mice	50–200 mg/kg (oral)	Increased cytokines, IgG, sIgA, and cecal lactobacilli.	(101)
CPPs	RAW264.7 macrophages	6.25–400 μg/mL (*in vitro*)	Enhanced NO and cytokine secretion; improved phagocytosis.	(81)
CPG	S180 tumor-bearing mice	50–100 mg/kg (oral)	Inhibited tumor growth; promoted humoral and cellular immunity.	(95)
CPW, CPS0.2/0.5/1	THP-1 cells	500 μg/mL (*in vitro*)	CPW anti-inflammatory; CPS series immunostimulatory (esp. CPS0.2).	(105)
CPPS	OVA-immunized mice	0.05–0.15 mg/mL (i.p.); 500 μg/mL (*in vitro*)	Increased lymphocyte proliferation and antibody levels.	(96)
CPPS, sCPPS	Splenic lymphocytes; OVA-immunized mice	0.196–12.5 μg/mL (*in vitro*); 0.05–0.15 mg/mL (s.c.)	sCPPs superior in T-cell activation and antibody production.	(23)
sCPPs	ND-vaccinated chickens	0.5–2.0 mg/mL (i.m.)	Enhanced lymphocyte proliferation and antibody titers.	(24)
CS-GO-CPP	RAW264.7 cells	0.78–12.5 μg/mL (*in vitro*)	Increased phagocytosis, NO, IL-4, IFN-γ, and NF-κB activation.	(22)
CPPS, sCPPS	Cyclophosphamide-immunosuppressed mice	Oral doses unspecified	Improved immune organs and immunoglobulin levels; sCPPS more effective.	(21)
sCPPS	Chicken T lymphocytes	0.391–6.25 μg/mL (*in vitro*)	Promoted T-cell proliferation and IL-2 expression.	(113)
pCPPS, CPPS	Duck hepatocytes; DHAV infection	4.88–39 μg/mL (*in vitro*)	pCPPS inhibited DHAV replication; CPPS inactive.	(25)
CPP	Ana-1 macrophages; mice	0.01–0.62 mg/g (oral/*in vitro*)	Dose-dependent TNF-α, IL-1β increase; enhanced immune organs.	(88)
CPP	Weaned piglets	1–2% dietary supplementation	Improved mucosal immunity (SIgA, IgG, IgM).	(109)
CPP	Laying hens	1.25–20 g/L (*in vitro*); 5–10 g/L (i.m.)	Antioxidant activity; increased ND antibody titers.	(108)
CPP	Weaned piglets	200–800 mg/kg (diet)	Improved growth, immunity, antioxidant capacity.	(112)
CPP	COPD model rats	50–200 mg/kg (oral); dexamethasone 1.95 mg/kg	Alleviated inflammation; modulated NF-κB/IκBα.	(106)
LCPP	TNF-α-stimulated HepG2 cells	1.25–25 mg/mL (*in vitro*)	Inhibited complement activation and C3 expression.	(104)
CPP	Chicken lymphocytes; ND-vaccinated hens	31–500 μg/mL (*in vitro*); 5–100 mg/kg (i.m.)	Enhanced lymphocyte proliferation and ND antibody titers.	(91)
CPPS-NE, CPPS	Hydrocortisone-immunosuppressed mice	300–900 mg/kg (oral)	Improved immunity; CPPS-NE superior to CPPS.	(37)
CPP	Cyclophosphamide-immunosuppressed Balb/c mice	250–1000 mg/kg (oral)	Increased Peyer’s patches, T-cell subsets, and IgA/IL-2/IFN-γ expression.	(92)

### 5.1 Regulation of immune cells

#### 5.1.1 Macrophages

**Figure 4:**
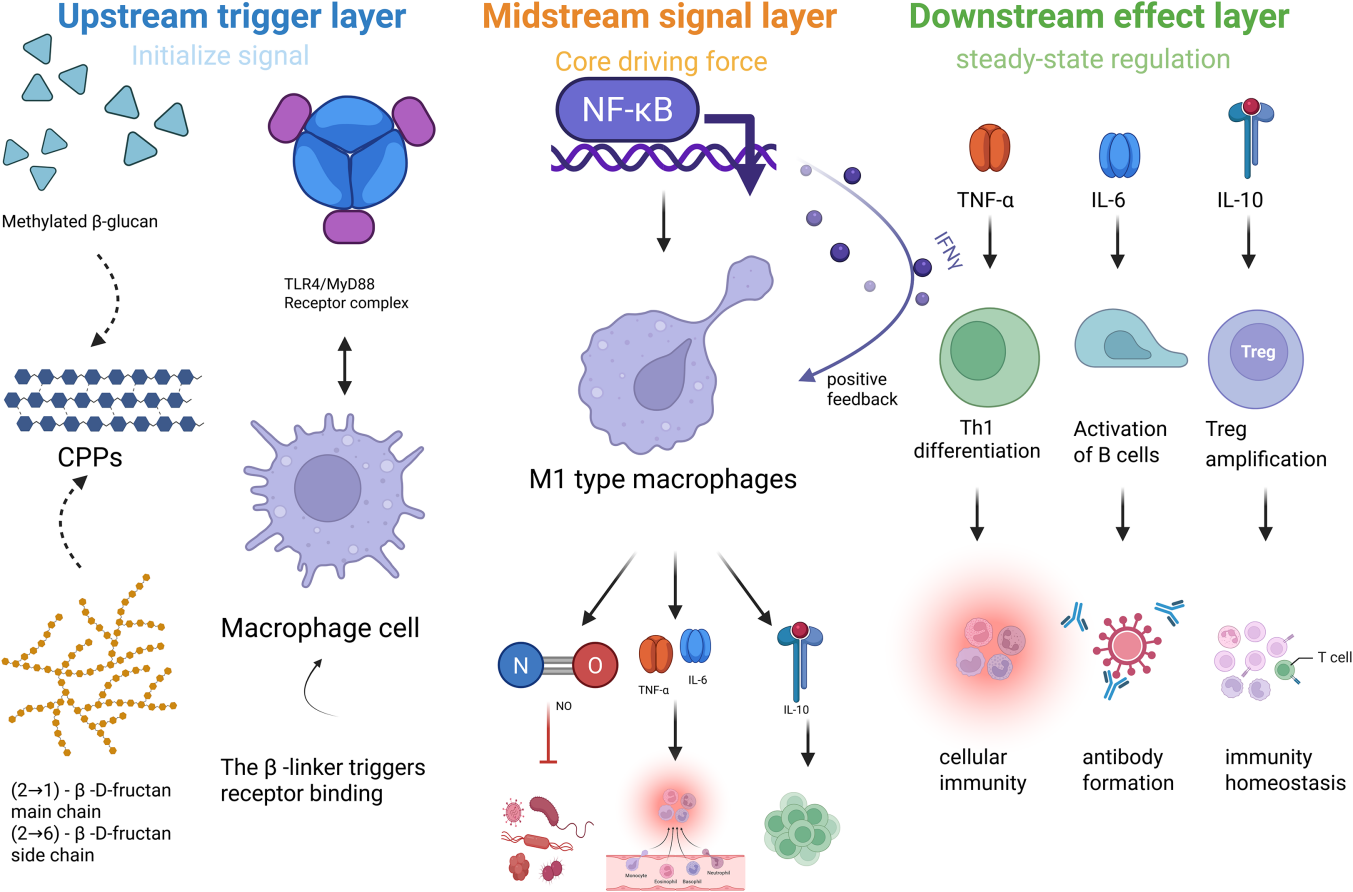
Macrophage-centric immunomodulatory mechanism of CPPs.

Macrophages are key components of the innate immune system. They possess the ability to phagocytose kill pathogens, as well as remove apoptotic cells (86). Through recognition of pathogen-associated molecular patterns, macrophages rapidly respond to infections by releasing cytokines such as interleukin-1 and tumor necrosis factor-alpha (TNF-α), thereby initiating inflammatory responses. In addition, as professional antigen-presenting cells, macrophages activate T cells, thus bridging innate and adaptive immunity while also participating in tissue repair and immune homeostasis (86, 87). The immunomodulatory effects of CPPs on macrophages are primarily mediated through receptor-dependent pathways. Notably, multiple studies have demonstrated that CPPs engage Toll-like receptor 4 (TLR4) on macrophage surfaces, triggering the Myeloid differentiation primary response 88 (MyD88)-dependent cascade and activating the NF-κB signaling pathway. This sequence constitutes the principal immunoregulatory axis by which CPPs initiate downstream cytokine production and adaptive immune modulation (**Figure 4**) (88). In addition to NF-κB activation, CPPs also modulate alternative signaling pathways. For example, Sun et al. reported that pectic polysaccharides RCAP-1 and RCAP-2 significantly promoted nitric oxide (NO) production in RAW264.7 macrophages, and mechanistic studies revealed that their activity was accompanied by enhanced phosphorylation of ERK and p38 MAPK, indicating that MAPK signaling contributes to CPP-mediated cytokine release in parallel with NF-κB (12). Likewise, Ji et al. found that a low-molecular-weight (<30 kDa) fructan fraction activated TLR4/MyD88 signaling while simultaneously triggering JNK and p38 pathways, thereby amplifying IL-6 and TNF-α secretion in a dose-dependent manner (81). Animal models further corroborate the macrophage-centered immunostimulatory role of CPPs. A compound preparation of CPPs significantly increased spleen indices and serum levels of granulocyte-macrophage colony-stimulating factor and TNF-α in BALB/c mice, primarily through M1 macrophage polarization and activation of the Stat1/Stat3 pathways (10). In melanoma-bearing C57BL/6 mice, the medium-molecular-weight (30–100 kDa) fraction CPPS-II enhanced the M1/M2 macrophage ratio, suppressed Stat3 phosphorylation, and synergized with chemotherapy to boost antitumor immunity (14). These results indicate that the STAT family is a critical target of CPP-mediated immune regulation, with Stat1 activation and Stat3 inhibition driving pro-inflammatory and antitumor phenotypes. To address the variability in reported molecular weights, we standardized the classification as follows: low-molecular-weight (<30 kDa), mid-molecular-weight (30–100 kDa), and high-molecular-weight (>100 kDa) fractions. While both low-MW β-(2→1)-fructans and mid-MW pectic polysaccharides (e.g., CPPS-II) exhibit macrophage-activating properties, their bioactivity likely arises from distinct structural motifs and receptor interactions (**Table 2**). Low-MW fructans primarily engage TLR4/MyD88 and MAPK signaling, whereas mid-MW pectins with RG-I domains contribute to M1 polarization via Stat1/Stat3 regulation. Collectively, these findings highlight that CPP-induced immunoregulation involves a coordinated network of NF-κB, MAPK, and STAT cascades that together fine-tune macrophage responses (12, 14, 81).

**Table 2:** Structural motifs of CPPs and their immunological activities.

Polysaccharide fraction	Key structural motifs	Dominant immune activity	Target cells/pathways	Rif.
RCAP-1	High GalA content, methyl-esterified pectic regions	Pro-inflammatory activation (NO, TNF-α, IL-6 secretion)	Macrophages via NF-κB and TLR4/MyD88	(12)
RCAP-2	High GalA content, methyl-esterified pectic regions	Pro-inflammatory activation (NO, TNF-α, IL-6 secretion)	Macrophages via NF-κB and TLR4/MyD88	(12)
CPPs	(2→1)-β-D-fructan backbone, (2→6)-β-D-fructan branches	Pro-inflammatory cytokine induction	Macrophages via TLR4/MyD88	(81)
CPPS-II	Medium-MW pectin-rich with RG-I domains	M1 macrophage polarization, anti-tumor immunity	Macrophages, STAT1/3	(14)
50WCP-II-I	Branched RG-I regions with GalA >60%	Complement inhibition, IL-10 induction	Complement pathway (C3), anti-inflammatory macrophages	(72)
100WCP-II-I	Branched RG-I regions with GalA >60%	Complement inhibition, IL-10 induction	Complement pathway (C3), anti-inflammatory macrophages	(72)
sCPPs	Sulfated glucan residues	Enhanced lymphocyte proliferation, antibody production	T and B lymphocytes	(24)
Se-CPPs	Selenoxide-modified pectin	Balanced Th1/Th2 cytokine upregulation	T lymphocytes	(23)
CS-GO-CPP	Nano-carrier conjugation with chitosan-graphene oxide	Enhanced phagocytosis, NF-κB activation	Macrophages	(22)

#### 5.1.2 T lymphocytes

T lymphocytes are the primary effector cells in adaptive immunity, recognizing and killing infected or tumor cells. According to their function, The cells can be mainly divided into T helper cells (Th cells) and cytotoxic T lymphocytes (CTLs) (89). Th cells secrete cytokines to stimulate the proliferation and differentiation of B cells and CTLs, regulating and amplifying the immune response, while CTLs induce the death of abnormal cells. T cells can also proliferate to form memory cells, which produce an efficient immune response upon re-infection with the same antigen (90). CPPs have exhibited remarkable immunomodulatory effects on T cells in multiple *in vitro* and *in vivo* studies. *In vitro*, CPPs could markedly enhance the proliferation of peripheral blood lymphocytes from chickens. The proliferation of CD4^+^ T cells was more pronounced than that of CD8^+^ T cells, and CPPs did not affect the proliferation of peripheral blood mononuclear cells from chickens. This indicates that CPPs may enhance cellular immunity, possibly by enhancing the proliferation and differentiation of Th cells (91). In immunosuppressed mice induced by cyclophosphamide (CP), low and medium doses of CPPs markedly increased the expression of CD3^+^, CD4^+^, and CD8^+^ T cell subsets in the small intestinal mucosa. However, high doses of CPPs exhibited a slight inhibitory effect. Furthermore, CPPs increased the thymus and spleen indices, promoting the development and maturation of T cells (92). CPPs can also regulate the differentiation of T cells in Peyer’s patches. CPPs inhibited the differentiation of CD8^+^ T cells and enhanced the differentiation of CD4^+^ T cells, which was reflected in the increased CD4^+^/CD8^+^ ratio and enhanced immune response (80).

In summary, CPPs can promote the proliferation and differentiation of T cells, as well as balance thereby enhancing adaptive immunity. These effects are closely related to the development and maturation of immune organs and the restructuring of the cytokine network.

#### 5.1.3 B lymphocytes

B lymphocytes mediate humoral immunity. B lymphocytes recognize antigens and differentiate into plasma cells, which secrete antibodies that can either inhibit a pathogen or facilitate its phagocytosis by another cell (93). Antibodies can also activate the complement system, providing feedback to the immune response. Few B cells differentiate into memory cells that rapidly divide in response to secondary infection with the same antigen (94). *C. pilosula* fructan (CPG) can significantly increase the B cell-mediated humoral immunity in S180 tumor-bearing mice. Tumor-specific immunoglobulin G (IgG) and immunoglobulin M (IgM) levels in the sera of mice injected with CPG were higher than those in the control group, which means that the B cell activity was enhanced, and the antibody secretion was increased. Thymus and spleen indices were increased. The proportion of CD19^+^ B cells in the spleen was increased in the CPG group. Mechanistically, CPG upregulated the expression of interleukin-2 (IL-2), interferon-γ (IFN-γ), and TNF-α to induce the proliferation and differentiation of B cells (95). CPPs demonstrated vigorous adjuvant activity in the vaccine model, greatly enhancing the immunogenicity of the vaccine and promoting B cell activation and antibody production (96). These results suggested that it is possible to use immune enhancement and antitumor therapy based on the regulation of B cells.

#### 5.1.4 Natural killer cells

NK cells are a crucial component of innate immunity. NK cells can kill virus-infected and tumor cells without prior antigenic stimulation (97). NK cells recognize the lack of expression of major histocompatibility complex class I molecules on target cells and induce cytolysis of target cells through the release of perforin, granzymes, and cytokines like IFN-γ, which can mediate the function of other cells (98). CPPs can enhance the activity of NK cells. In BALB/c mice, the medium and high doses of CPPs can enhance the cytotoxicity of NK cells and induce the maturation and function recovery of immune cells (10). It offers an explorative basis for the study of new immunomodulators.

### 5.2 Modulation of immune organs

There are two types of immune organs: central immune organs (such as the bone marrow and thymus) and peripheral immune organs (including the spleen and lymph nodes). The Immune organ index is the ratio of the weight of the immune organ and the body weight (BW). It can reflect the development and function of the immune organ (an important indicator of immune function) (99). In addition, the immune organs can also secrete cytokines, such as IL-2, IL-6, IFN-γ, and TNF-α. These cytokines can induce the activation, proliferations, and differentiation of immune cells and are involved in both inflammatory and anti-infection processes. It is very essential to ensure structure and function of the immune organ to maintain the immune homeostasis, prevent pathogen invasion,and induce the correct response to vaccines (100).

CPPs have strong protective and regulatory effects in the CP-induced immunosuppressive mouse model. In the present experiment, the mice were injected intraperitoneally with CP at 60 mg/kg·BW·d for three consecutive days and then administered different doses of CPPs (50, 100, 200 mg/kg/day) orally for 7 days. The spleen index of all groups was significantly increased compared to the model group, and the spleen index of the 100 mg/kg/day group was even higher than that of the standard control group. The thymus index of the 200 mg/kg/day group was significantly increased and approached the normal group. In addition, the liver index of the 100 and 200 mg/kg/day groups was significantly increased compared with the model group (101). These findings indicate that CPPs effectively alleviate CP-induced damage to immune organs, particularly improving spleen atrophy, and also exert protective and enhancing effects on thymus and liver-related immune functions. CPPs may improve immunity by increasing immune organ indices and relieving immune organ suppression, providing experimental evidence for their potential as natural immunomodulators.

### 5.3 Modulation of immune factors

Immune factors are important molecules that mediate the regulation of the immune system, such as cytokines, antibodies, and complement. Cytokines include interleukins, interferons and tumor necrosis factors (102). They can mediate the proliferation, differentiation and activation of immune cells. They are involved in both innate and adaptive immune responses. Antibodies are secreted by B cells to mediate the neutralization of target cells, enhance phagocytosis of target cells, and induce antibody-dependent cellular cytotoxicity. Complements can directly cause damage to the pathogen’s membrane and cause inflammation. They can also enhance the effects of the antibody (103).

CPPs possess multidimensional pharmacological effects on regulating immune factors. On one hand, CPPs significantly regulate the secretion of multiple cytokines with bidirectional modulation. For example, low-molecular-weight (<30 kDa) β-(2→1)-fructan-rich fractions have been shown to bind TLR4 and activate MyD88-dependent NF-κB signaling pathways, resulting in increased production of pro-inflammatory cytokines such as TNF-α and IL-6, which are beneficial for applications in vaccine adjuvants and tumor immunotherapy (81). Conversely, pectic polysaccharides enriched in rhamnogalacturonan-I regions with high galacturonic acid content (>60%) exhibit complement C3 inhibitory activity (CH_50_=2.06 mg/mL) and upregulate anti-inflammatory cytokine IL-10, suggesting potential utility in autoimmune and chronic inflammatory diseases such as COPD (104). When CPPs were applied to lipopolysaccharide-stimulated THP-1 inflammatory models, CPPs suppressed the expression of pro-inflammatory cytokines TNF-α and IL-1β while enhancing the activity of immune function in non-stimulated THP-1 cells, indicating that CPPs have the potential to balance the expression of immune factors (105). When applied to the chronic obstructive pulmonary disease model, CPPs downregulated the expression of pro-inflammatory cytokines (TNF-α, IL-6, IL-1β) and upregulated the expression of the anti-inflammatory cytokine IL-10 by suppressing the NF-κB signaling pathway, thereby reducing the expression of pro-inflammatory cytokines and alleviating airway inflammation (106). When applied to CP-induced immunosuppressed mice, CPPs significantly increased the contents of IL-2, IL-6 and TNF-α in serum and enhanced the immune response (107). When applied to poultry models, CPPs increased the titers of Newcastle disease virus antibodies, indicating that CPPs can enhance the humoral immune response and improve the vaccine effectiveness (108). When applied to piglets, CPPs significantly upregulated the protein and mRNA expression of SIgA, IgG and IgM in the small intestinal mucosa, indicating that CPPs can participate in local intestinal immunity (109).

Emerging evidence suggests that gut microbiota modulation may represent a primary mechanism underlying the systemic immunoregulatory effects of CPPs. Specifically, CPPs have been shown to enrich butyrate-producing bacterial taxa, leading to increased production of short-chain fatty acids (SCFAs) such as butyrate. Butyrate acts as a ligand for G-protein-coupled receptor 43 (GPR43), a receptor expressed on colonic epithelial cells and immune cells. Engagement of GPR43 can drive the differentiation and expansion of regulatory T cells (Tregs), thereby contributing to mucosal and systemic immune tolerance (9). From a translational perspective, it remains to be elucidated whether gut microbiota modulation constitutes the dominant axis of CPPs immunoregulation *in vivo* or acts in parallel with direct immune cell binding. Comparative studies evaluating germ-free versus conventional animal models could clarify the relative contributions of microbiota-dependent and microbiota-independent mechanisms.

### 5.4 Modulation of the complement system

Complement is an essential component of innate immunity (110). It consists of some plasma proteins and can be activated by the classic, alternative, and lectin pathways. After activation, complement can directly lyse pathogens and help phagocytes eliminate antigens by releasing active fragments, such as C3a and C5a and attracting immune cells to initiate an inflammatory response. Moreover, it synergizes with antibodies to amplify adaptive immunity and plays irreplaceable roles in infection control, immune surveillance, and maintaining immune balance (111).

Research has shown that CPPs possess definitive regulatory effects on the complement system, with anti-complement activity confirmed in several studies. For example, Lu CPPs significantly inhibit the activation of both classical and alternative complement path. The half-maximal inhibitory concentration (CH_50_) for the classical pathway was 2.061 ± 0.127 mg/mL, and for the alternative pathway (AP_50_) it was 6.725 ± 0.895 mg/mL, with effects showing explicit dose dependency. Mechanistic studies revealed that Lu CPPs significantly suppressed complement C3 expression in TNF-α-stimulated human hepatoma HepG2 cells. With increasing LCPP concentrations, C3 mRNA levels declined in a dose-dependent manner, suggesting inhibition of inflammation-mediated complement activation (104). Additionally, pectic polysaccharides 50WCP-II-I and 100WCP-II-I, extracted from *C. pilosula* roots, demonstrated complement-fixing activity. Enzymatic hydrolysis showed that activity was primarily mediated by branched regions, with high molecular weight hydrolysates (e.g., 50WCP-II-Ia and 100WCP-II-Ia) exhibiting greater activity than the native polysaccharides (72).

*In vivo* studies have also shown that CPPs positively regulate immune parameters. For instance, CPPs significantly increased serum levels of immunoglobulins (IgA, IgM, IgG) and complement components C3 and C4 in weaned piglets, thereby enhancing overall immune function (112). These findings provide pharmacological evidence for the use of CPPs in treating diseases associated with complement overactivation, including autoimmune disorders.

## 6 Structural modification and immunoactivity

In recent years, structural modification of CPPs to enhance their immunomodulatory activities has received considerable attention. Physical or chemical modification can significantly increase the water solubility, stability and bioavailability of CPPs, thereby enhancing their immune function and facilitating the development of new immunostimulants.

**Figure 5:**
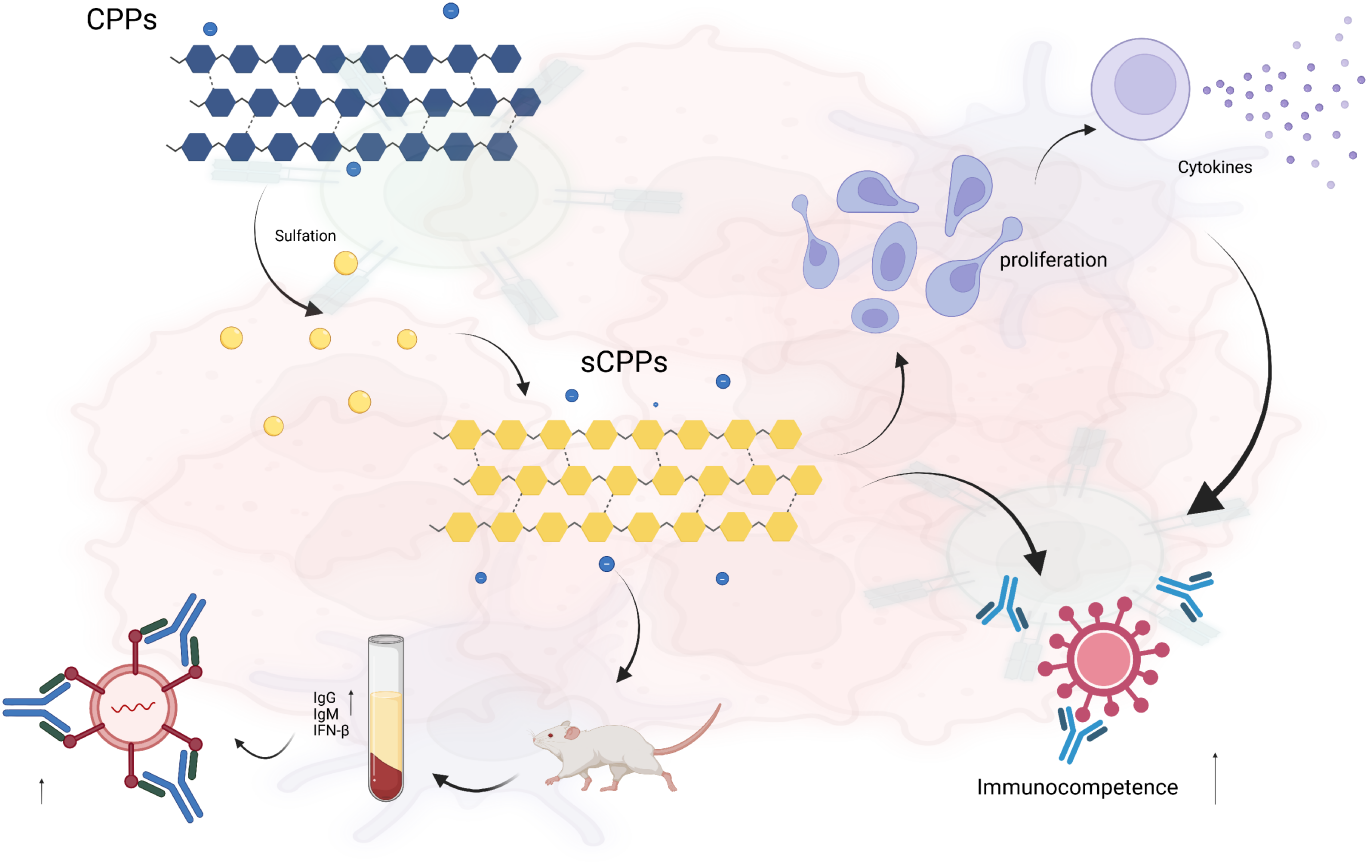
Immune regulatory mechanisms of sCPPs.

Sulfation is the most commonly reported method of modification of polysaccharides. Previous reports have demonstrated that sulfated *C. pilosula* polysaccharides [sCPPs; (−) Configuration, 1→6-β-glucan, degree of sulfation (DS) 0.26–0.32] exhibited enhanced immunoactivity (113). Due to the presence of additional negative charges and spatial structures, sCPPs can bind more strongly to receptors on immune cells and activate the immune response. Our results showed that sCPPs can significantly promote the proliferation of lymphocytes and the secretion of cytokines. When co-stimulated with phytohemagglutinin-P, sCPPs exhibited higher T lymphocyte proliferation than unmodified CPPs, which proved that sCPPs had enhanced T-cell mediated immunity. In addition, sCPPs could significantly increase the levels of IgG and IgM in the serum of mice, upregulate the expression level of the interferon-β (IFN-β) gene, and enhance antiviral immunity (**Figure 5**) (24).

Phosphorylation is the most frequently reported polysaccharide modification strategy. Phosphorylated *C. pilosula* polysaccharides (pCPPs) showed apparent antiviral activity. As shown in (25), pCPPs could markedly inhibit DHAV infection, enhance the survival rate of duck embryo hepatocytes,and decrease the titers of viral replication and particles. Meanwhile, pCPPs could attenuate DHAV-induced IFN-β expression, indicating that they may inhibit viral replication to exert their anti-inflammatory activity. The introduction of phosphate groups enhanced the negative charge and spatial complexity of CPPs, and increasing the interactions between CPPs and viruses, as well as phagocytic cells, and significantly enhanced the immunoactivity of CPPs.

**Figure 6:**
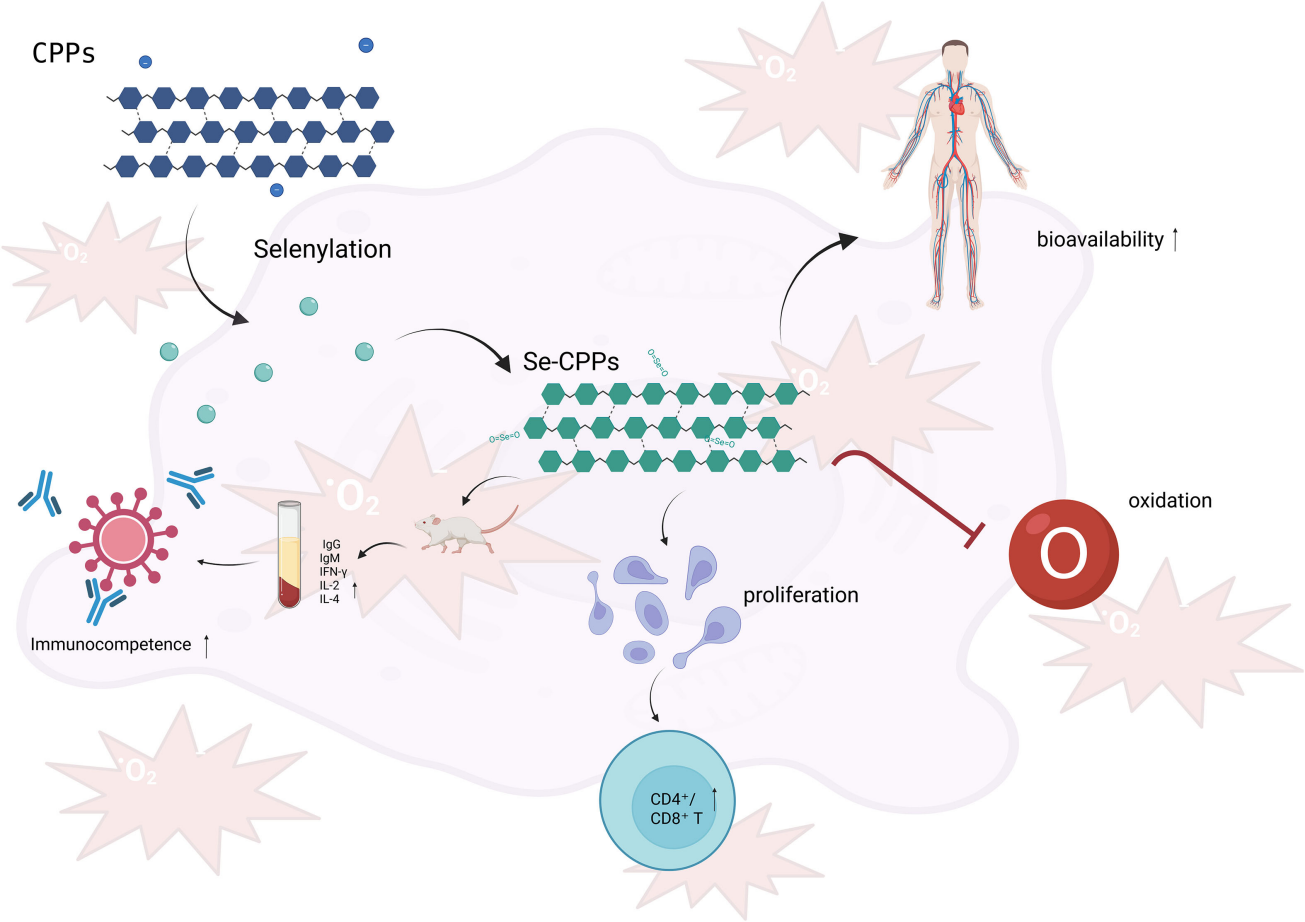
Immune regulatory mechanisms of Se-CPPs.

As a newly developed modification strategy, selenization showed great potential in immunoenhancement. Selenium is an essential trace element which has been reported to possess specific antioxidant and immunomodulatory activities. As shown in (23), the immunomodulatory activity of selenium-modified CPPs (Se-CPPs) was significantly more potent than that of CPPs. *In vitro*, Se-CPPs could dramatically enhance the proliferation of lymphocytes and increase the CD4^+^/CD8^+^ T cell ratio, reflecting an improved balance of the immune system. *In vivo*, the serum levels of IgG, IgM, IFN-γ, IL-2, and IL-4 in mice administered with Se-CPPs were increased, and the mice exhibited higher indices of immune organ and immune cell function. The formation of a novel quaternary structure of CPPs (selenoxide bond O=Se=O) endowed CPPs with new biological functions and enhanced the antioxidant property and bioavailability of CPPs (**Figure 6**).

Nanotechnology offers new opportunities to enhance the immunostimulatory properties of CPPs. For example, a nanocomposite of CPPs and chitosan-graphene oxide (CS-GO-CPP) exhibited enhanced macrophage-activating properties. The results revealed that CS-GO-CPP enhanced the phagocytosis of RAW264.7 macrophages, increased NO production, elevated the secretion of IL-4 and IFN-γ, and upregulated costimulatory molecules, including CD40, CD86, and F4/80 (22). Further mechanistic studies revealed that CS-GO-CPP activates the NF-κB pathway to upregulate the expression of NF-κB and promote the nuclear translocation of p65, thereby enhancing macrophage immune responses. In addition, nano-carriers can further improve the stability and *in vivo* retention of CPPs, as well as targeting and cellular uptake, which greatly enhances the immunoregulatory effects.

Comparatively, different modification strategies exhibit distinct immunological advantages. Sulfation generally enhances the negative charge density of CPPs, improving their recognition by immune cell surface receptors and resulting in stronger lymphocyte proliferation and antibody production, which makes it particularly effective for vaccine adjuvant applications (24). Phosphorylation, on the other hand, has shown pronounced antiviral effects by attenuating viral replication and modulating type I interferon responses, suggesting its potential utility in infectious disease models (25). Selenization tends to produce the most profound systemic effects, not only enhancing T and B cell activity but also balancing Th1/Th2 cytokine secretion, thereby improving overall immune homeostasis and antioxidant capacity (23). Nanocarrier-based modifications are unique in that they do not directly alter polysaccharide chemistry but rather improve stability, bioavailability, and targeted delivery, thus complementing chemical modifications (22). Taken together, sulfation may be optimal for adjuvanticity, phosphorylation for antiviral interventions, and selenization for systemic immunoenhancement, while nanotechnology offers an important auxiliary strategy to maximize efficacy.

In summary, structural modification strategies, including sulfation, phosphorylation, selenization, and the introductionof nanocarriers, can be used to enhance the immunostimulatory activity of CPPs. These new and diverse strategies provide a solid foundation for the further development and functional expansion of CPPs in the field of immune modulation.

## 7 Conclusion and future prospects

As one of the primary active components of *C. pilosula*, CPPs exhibit diverse morphologies, a wide range of sources, and remarkable bioactivities (114–116). In recent years, CPPs have gained increasing attention in research focusing on their extraction methods, structural characteristics, immunoregulatory effects, and structural modification strategies, providing a comprehensive understanding of their immunomodulatory properties and translational potential. Notably, macrophage activation appears to be the primary immunoregulatory axis through which CPPs exert their wide-ranging effects, serving as the initiating signal for the modulation of adaptive immunity and systemic immune homeostasis. Currently, extraction technologies for CPPs have evolved from traditional HWE to various modern and environmentally friendly methods, including EAE, UAE, MAE, and SWE (49, 81, 117). These advancements have significantly improved both extraction efficiency and the retention of bioactive components. Further refinement through multi-step gradient ethanol precipitation, IEC, and GPC provide a solid foundation for structural elucidation and functional studies of CPPs (118, 119).

Structurally, CPPs are predominantly heterogeneous pectic polysaccharides, mainly composed of monosaccharides such as GalA, Gal, Ara, and Rha. The complex linkage patterns between the backbone and side chains are closely associated with their biological activity (120, 121). In terms of immunomodulation, CPPs exhibit multifaceted and multi-targeted immunoenhancing effects, including macrophage activation, promoting the proliferation and differentiation of T and B lymphocytes, enhancing NK cell functions, improving immune organ indices, and regulating cytokines and the complement system (72, 80, 96). Their mechanisms of action involve the activation or inhibition of key signaling pathways, including NF-κB, TLR4/MyD88, and STAT (signal transducer and activator of transcription), showcasing their broad-spectrum immunoregulatory potential. Additionally, CPPs hold promise for applications in adjuvant development, anti-tumor immunity, and intervention in immunosuppressive disorders (81, 106).

Structural modification has emerged as a powerful strategy for enhancing the immunological activity of CPPs. Chemical modifications, including sulfation, phosphorylation, and selenization, significantly improve the binding affinity of CPPs to immune cell surface receptors, thereby boosting immune response efficiency (23–25). Furthermore, nanocarrier-based systems enhance the stability and targeting ability of CPPs, expanding their applicability in immunotherapy (22).

Despite these advancements, several challenges remain. First, the structural complexity of CPPs limits the comprehensive analysis of their structure-activity relationships. There is an urgent need for high-throughput, fine-resolution structural characterization techniques, and a standardized nomenclature system. Second, research on CPPs’ mechanisms is primarily at the cellular and animal levels, with limited clinical validation. Their safety profile, metabolic pathways, and long-term effects require systematic evaluation. Furthermore, the precise regulatory mechanisms of CPPs in specific immune-related diseases, such as autoimmune disorders and tumor immune evasion, remain unclear, hindering their application in precision medicine.

Future research directions include:

1. Strengthening the detailed structural elucidation of CPPs and establishing a structure-function database to support further understanding of their immunological mechanisms.
2. Integrating systems biology and multi-omics technologies to uncover the dynamic regulatory roles of CPPs within *in vivo* immune networks.
3. Promoting standardized extraction and formulation development to facilitate the translational leap from basic research to clinical applications.
4. Conducting larger-scale and higher-quality preclinical and clinical studies to validate the efficacy and safety of CPPs in areas such as vaccine adjuvants, immunodeficiencies, and cancer immunotherapy.

In conclusion, CPPs, as naturally derived immunoreactive polysaccharides, hold significant promise for advancing immunological research and developing innovative functional foods and biopharmaceutical applications. Through interdisciplinary collaboration and technological innovation, CPPs are expected to bridge the gap from “laboratory discovery” to “clinical application” in the field of immune intervention.

## References

[1] Gong Y Hu R Yue X. Study on the origins and historical sources of codonopsis and its clinical application research progress. J Chin Tradit Med. (2024), 1–10.

[2] GBIF, global biodiversity information facility. Available online at: https://www.gbif.org/.

[3] Pharmacopoeia of the People’s Republic of China. Available online at: https://ydz.chp.org.cn//main.

[4] Zhang H Li C Ji C. Research progress on the chemical constituents, pharmacological effects, and applications of codonopsis as a medicinal and edible herb. Food Sci. (2024) 45:338–48.

[5] Zhang C Yu M Chen R Sun X. Research progress on the pharmacological effects of Codonopsis pilosula. Chin New Drugs Clin Pharmacol. (2024) 35:765–70. doi: 10.19378/j.issn.1003-9783.2024.05.019

[6] Yin L Yin C Lu C Zeng S Wang H. Active components of Codonopsis pilosula and their applications in livestock and poultry production. Feed Res. (2023) 46:155–8. doi: 10.13557/j.cnki.issn1002-2813.2023.11.032

[7] Wang B Li L Yang X Wang T Zhang W. Research and progress on extraction of main active components from Codonopsis pilosula. Inf Anim Husbandry Vet Sci Technol. (2023) 08):42–5.

[8] Luan F Ji Y Peng L Liu Q Cao H Yang Y. Extraction, purification, structural characteristics and biological properties of the polysaccharides from Codonopsis pilosula: A review. Carbohydr Polym. (2021) 261. doi: 10.1016/j.carbpol.2021.117863, PMID: 33766352

[9] Rong X Shu Q. Modulating butyric acid-producing bacterial community abundance and structure in the intestine of immunocompromised mice with neutral polysaccharides extracted from Codonopsis pilosula. Int J Biol Macromol. (2024) 278:134959. doi: 10.1016/j.ijbiomac.2024.134959, PMID: 39179083

[10] Peng Y Song Y Wang Q Hu Y He Y Ren D. *In vitro* and *in vivo* immunomodulatory effects of fucoidan compound agents. Int J Biol Macromol. (2019) 127:48–56. doi: 10.1016/j.ijbiomac.2018.12.197, PMID: 30593813

[11] Bai R Li W Li Y Ma M Wang Y Zhang J. Cytotoxicity of two water-soluble polysaccharides from Codonopsis pilosula Nannf. var. modesta (Nannf.) L.T.Shen against human hepatocellular carcinoma HepG2 cells and its mechanism. Int J Biol Macromol. (2018) 120:1544–50. doi: 10.1016/j.ijbiomac.2018.09.123, PMID: 30248423

[12] Sun QL Li YX Cui YS Jiang SL Dong CX Du J. Structural characterization of three polysaccharides from the roots of Codonopsis pilosula and their immunomodulatory effects on RAW264.7 macrophages. Int J Biol Macromol. (2019) 130:556–63. doi: 10.1016/j.ijbiomac.2019.02.165, PMID: 30831168

[13] Li J Zhang X Cao L Ji J Gao J. Three Inulin-Type Fructans from Codonopsis pilosula (Franch.) Nannf. Roots and Their Prebiotic Activity on Bifidobacterium longum. Molecules. (2018) 23. doi: 10.3390/molecules23123123, PMID: 30501018 PMC6320984

[14] Li N Xiong YX Ye F Jin B Wu JJ Han MM. Isolation, purification, and structural characterization of polysaccharides from Codonopsis pilosula and their anti-tumor bioactivity by immunomodulation. Pharm (Basel). (2023) 16. doi: 10.3390/ph16060895, PMID: 37375842 PMC10303390

[15] Li N Yang C Xia J Wang W Xiong W. Molecular mechanisms of Codonopsis pilosula in inhibiting hepatocellular carcinoma growth and metastasis. Phytomedicine. (2024) 128:155338. doi: 10.1016/j.phymed.2024.155338, PMID: 38520835

[16] Yexia C Lili G Qiao D Xin Z. The response surface methodology (RSM) was used to optimize the ultrasonic-assisted extraction of Codonopsis pilosula polysaccharides and evaluate their biological activity. Mol Plant Breeding. (2018) 16:7495–502. doi: 10.13271/j.mpb.016.007495

[17] Yu L Chen H Lou F. Optimization of microwave-assisted extraction process for Luolong codonopsis polysaccharides. Food Res Dev. (2011) 32:26–9.

[18] Gao J Zhu X Song K Liu X Chen H Shi T. Optimization of the extraction process of polysaccharides from codonopsis using composite enzyme assistance. Chin Proprietary Med. (2018) 40:1189–93.

[19] Ji R Wang Z Kuang H. Extraction, purification, structural characterization, and biological activity of polysaccharides from Schisandra chinensis: A review. Int J Biol Macromol. (2024) 271. doi: 10.1016/j.ijbiomac.2024.132590, PMID: 38788881

[20] Tang Z Huang G. Extraction, structure, and activity of polysaccharide from Radix astragali. Biomed Pharmacother. (2022) 150. doi: 10.1016/j.biopha.2022.113015, PMID: 35468585

[21] Hao Y Nie C Wu X Liu C Hao X. Research progress on the immunomodulatory effects of codonopsis polysaccharides and their structural modification. China Pharm Guide. (2018) 15:25–8.

[22] Sun M Ren Z Wei T Huang Y Zhang X Zheng Q. Preparation, characterization and immune activity of Codonopsis pilosula polysaccharide loaded in chitosan-graphene oxide. Int J Biol Macromol. (2022) 221:1466–75. doi: 10.1016/j.ijbiomac.2022.08.209, PMID: 36070821

[23] Gao Z Zhang C Jing L Feng M Li R Yang Y. The structural characterization and immune modulation activitives comparison of Codonopsis pilosula polysaccharide (CPPS) and selenizing CPPS (sCPPS) on mouse *in vitro* and vivo. Int J Biol Macromol. (2020) 160:814–22. doi: 10.1016/j.ijbiomac.2020.05.149, PMID: 32446900

[24] Zhao X Hu Y Wang D Liu J Guo L. The comparison of immune-enhancing activity of sulfated polysaccharidses from Tremella and Condonpsis pilosula. Carbohydr Polym. (2013) 98:438–43. doi: 10.1016/j.carbpol.2013.06.043, PMID: 23987365

[25] Ming K Chen Y Yao F Shi J Yang J Du H. Phosphorylated Codonopsis pilosula polysaccharide could inhibit the virulence of duck hepatitis A virus compared with Codonopsis pilosula polysaccharide. Int J Biol Macromol. (2017) 94:28–35. doi: 10.1016/j.ijbiomac.2016.10.002, PMID: 27713010

[26] Yu Y Shen M Song Q Xie J. Biological activities and pharmaceutical applications of polysaccharide from natural resources: A review. Carbohydr Polym. (2018) 183:91–101. doi: 10.1016/j.carbpol.2017.12.009, PMID: 29352896

[27] Nai J Zhang C Shao H Li B Li H Gao L. Extraction, structure, pharmacological activities and drug carrier applications of Angelica sinensis polysaccharide. Int J Biol Macromol. (2021) 183:2337–53. doi: 10.1016/j.ijbiomac.2021.05.213, PMID: 34090852

[28] Yu X Miao Z Zhang L Zhu L Sheng H. Extraction, purification, structure characteristics, biological activities and pharmaceutical application of Bupleuri Radix Polysaccharide: A review. Int J Biol Macromol. (2023) 237. doi: 10.1016/j.ijbiomac.2023.124146, PMID: 36965565

[29] Gong H Gan X Li Y Chen J Xu Y Shi S. Review on the genus Polygonatum polysaccharides: Extraction, purification, structural characteristics and bioactivities. Int J Biol Macromol. (2023) 229:909–30. doi: 10.1016/j.ijbiomac.2022.12.320, PMID: 36608864

[30] Liu Y Shi Y Zou J Zhang X Zhai B Guo D. Extraction, purification, structural features, biological activities, modifications, and applications from Taraxacum mongolicum polysaccharides: A review. Int J Biol Macromol. (2024) 259:129193. doi: 10.1016/j.ijbiomac.2023.129193, PMID: 38191106

[31] Chen R Zhou X Deng Q Yang M Li S Zhang Q. Extraction, structural characterization and biological activities of polysaccharides from mulberry leaves: A review. Int J Biol Macromol. (2024) 257. doi: 10.1016/j.ijbiomac.2023.128669, PMID: 38092124

[32] Chen H Wen Y Yu Z Du X Pan W Liu T. Codonopsis pilosula polysaccharide alleviates rtenone-induced murine brain organoids death through downregulation of gene body DNA methylation modification in the ZIC4/PGM5/CAMTA1 axis. Biochem Biophys Rep. (2024) 37:101593. doi: 10.1016/j.bbrep.2023.101593, PMID: 38074999 PMC10698575

[33] Wang Y Zhao Y Chen H Lu T Yang R Weng X. Effect of Codonopsis pilosula polysaccharide on the quality of sheep semen preservation at 4 degrees C. Anim Biosci. (2024) 37:1001–6. doi: 10.5713/ab.23.0258, PMID: 38271972 PMC11065951

[34] Feng G Zhang XF. Production of a codonopsis polysaccharide iron complex and evaluation of its properties. Int J Biol Macromol. (2020) 162:1227–40. doi: 10.1016/j.ijbiomac.2020.06.210, PMID: 32615228

[35] Ma W Ji Y. Research progress on the extraction, purification, structural identification, and biological activity of plant polysaccharides. Grain Sci Technol Econ. (2019) 44:77–80. doi: 10.16465/j.gste.cn431252ts.20190717

[36] Wang Y Wang C Xue H Jin Y Yang M Leng F. Comparative analysis of three kinds of extraction kinetic models of crude polysaccharides from Codonopsis pilosula and evaluate the characteristics of crude polysaccharides. Biomass Convers Biorefin. (2022) 19:1–17. doi: 10.1007/s13399-022-02518-w, PMID: 35342681 PMC8933859

[37] Shi F Chen Y Zhou C Yu S. Optimization of codonopsis polysaccharide extraction process and antioxidant activity study using response surface methodology. Anim Husbandry Vet Med. (2021) 53:54–60.

[38] Fu YP Li LX Zhang BZ Paulsen BS Yin ZQ Huang C. Characterization and prebiotic activity *in vitro* of inulin-type fructan from Codonopsis pilosula roots. Carbohydr Polym. (2018) 193:212–20. doi: 10.1016/j.carbpol.2018.03.065, PMID: 29773375

[39] Li Q Hu D Zhang X Zhu R. Optimization of the extraction and purification process of codonopsis polysaccharides and its composition study. Chin Herbal Med. (2016) 47:2663–7.

[40] Meng H Zhao X He F Zhang W. Optimization of codonopsis polysaccharide extraction process and comparison of polysaccharide content in codonopsis from Lanzhou and Dingxi. Shizhen J Tradit Chin Med Pharmacol. (2020) 31:318–20.

[41] Leong YK Yang FC Chang JS. Extraction of polysaccharides from edible mushrooms: Emerging technologies and recent advances. Carbohydr Polym. (2021) 251:117006. doi: 10.1016/j.carbpol.2020.117006, PMID: 33142573

[42] Yang J Song Y Yu Y Yang X Zhang X Zhang W. Research progress on extraction techniques, structure-activity relationship, and biological functional mechanism of berry polysaccharides: A review. Int J Biol Macromol. (2024) 282:137155. doi: 10.1016/j.ijbiomac.2024.137155, PMID: 39505177

[43] Liang Q Han D Jiang J Yan G Kong L Sun H. Extraction, structural characteristics, bioactivities and application of polysaccharides from Acanthopanax senticosus (Rupr. Maxim.) harms: A review. Int J Biol Macromol. (2025) 299:139972. doi: 10.1016/j.ijbiomac.2025.139972, PMID: 39826744

[44] Wang T Chen Q Zou L Qin L Zhu H. Optimization of Codonopsis polysaccharide extraction process and comparison of polysaccharide content in codonopsis from different purchasing locations. J Southwest Normal University. (2016) 41:41–5. doi: 10.13718/j.cnki.xsxb.2016.02.009

[45] Wang T Ying X Zhang Q Xu Y Jiang C Shang J. Evaluation of the effect of ultrasonic pretreatment on the drying kinetics and quality characteristics of Codonopsis pilosula slices based on the grey correlation method. Molecules. (2023) 28. doi: 10.3390/molecules28145596, PMID: 37513468 PMC10385178

[46] Wang Z Zhu S Huang Y Chen C Guo P Xiang L. Optimization of the extraction process of pomegranate seed cake polysaccharides assisted by cellulase and its kinetic and thermodynamic study. Chin Proprietary Med. (2024) 46:3230–5.

[47] Nadar SS Rao P Rathod VK. Enzyme assisted extraction of biomolecules as an approach to novel extraction technology: A review. Food Res Int. (2018) 108:309–30. doi: 10.1016/j.foodres.2018.03.006, PMID: 29735063

[48] Ke L Duan X Cui J Song X Ma W Zhang W. Research progress on the extraction technology and activity study of Epimedium polysaccharides. Carbohydr Polym. (2023) 306:120602. doi: 10.1016/j.carbpol.2023.120602, PMID: 36746589

[49] Fan J Bai R Wang Y Yan Q Zhang Q Hu F. Extraction of polysaccharides from fresh codonopsis fermented by yeast and its immunological activity study. Modern Chin J Appl Pharmacol. (2022) 39:2444–50. doi: 10.13748/j.cnki.issn1007-7693.2022.19.003

[50] Gharibzahedi SMT Marti-Quijal FJ Barba FJ Altintas Z. Current emerging trends in antitumor activities of polysaccharides extracted by microwave- and ultrasound-assisted methods. Int J Biol Macromol. (2022) 202:494–507. doi: 10.1016/j.ijbiomac.2022.01.088, PMID: 35045346

[51] Mirzadeh M Arianejad MR Khedmat L. Antioxidant, antiradical, and antimicrobial activities of polysaccharides obtained by microwave-assisted extraction method: A review. Carbohydr Polym. (2020) 229:115421. doi: 10.1016/j.carbpol.2019.115421, PMID: 31826454

[52] Ali MS Haq M Park SW Han JM Kim JW Choi MS. Recent advances in recovering bioactive compounds from macroalgae and microalgae using subcritical water extraction: Prospective compounds and biological activities. Food Chem. (2025) 469:142602. doi: 10.1016/j.foodchem.2024.142602, PMID: 39724698

[53] Rodrigues Barbosa J Dos Santos Freitas MM da Silva Martins LH de Carvalho RNJ. Polysaccharides of mushroom Pleurotus spp.: New extraction techniques, biological activities and development of new technologies. Carbohydr Polym. (2020) 229:115550. doi: 10.1016/j.carbpol.2019.115550, PMID: 31826512

[54] Zhang R Zhang X Liu J Shu J Liu H Chen Q. Optimization of subcritical water extraction process for codonopsis. J Exp Prescriptions Tradit Chin Med. (2013) 19:34–7.

[55] Segaran A Chua LS. Review of recent applications and modifications of aqueous two-phase system for the separation of biomolecules. Int J Biol Macromol. (2024) 276:133856. doi: 10.1016/j.ijbiomac.2024.133856, PMID: 39009267

[56] Ahmed T Yamanishi C Kojima T Takayama S. Aqueous two-phase systems and microfluidics for microscale assays and analytical measurements. Annu Rev Anal Chem (Palo Alto Calif). (2021) 14:231–55. doi: 10.1146/annurev-anchem-091520-101759, PMID: 33950741

[57] Lu S Gu W Ma Q Tian R Qiu R Ma L. Extraction, structural characterization, and biological activities of a new glucan from Codonopsis pilosula. Sci Rep. (2023) 13:4504. doi: 10.1038/s41598-023-31660-2, PMID: 36934161 PMC10024767

[58] Lai JY Fan XL Zhang HB Wang SC Wang H Ma X. Polygonum cuspidatum polysaccharide: A review of its extraction and purification, structure analysis, and biological activity. J Ethnopharmacol. (2024) :331:118079. doi: 10.1016/j.jep.2024.118079, PMID: 38513776

[59] Mo X Guo D Jiang Y Chen P Huang L. Isolation, structures and bioactivities of the polysaccharides from Radix Hedysari: A review. Int J Biol Macromol. (2022) 199:212–22. doi: 10.1016/j.ijbiomac.2021.12.095, PMID: 34995662

[60] Wang M Tang H-P Bai Q-X Yu A-Q Wang S Wu L-H. Extraction, purification, structural characteristics, biological activities, and applications of polysaccharides from the genus Lilium: A review. Int J Biol Macromol. (2024) 267. doi: 10.1016/j.ijbiomac.2024.131499, PMID: 38614164

[61] Xue T Ruan K Tang Z Duan J Xu H. Isolation, structural properties, and bioactivities of polysaccharides from Althaea officinalis Linn.: A review. Int J Biol Macromol. (2023) 242. doi: 10.1016/j.ijbiomac.2023.125098, PMID: 37245776

[62] Zhang Z-J Hu W-J Yu A-Q Wu L-H Yang D-Q Kuang H-X. Review of polysaccharides from Chrysanthemum morifolium Ramat.: Extraction, purification, structural characteristics, health benefits, structural-activity relationships and applications. Int J Biol Macromol. (2024) 278. doi: 10.1016/j.ijbiomac.2024.134919, PMID: 39179070

[63] Zou Y-F Zhang Y-Y Paulsen BS Fu Y-P Huang C Feng B. Prospects of Codonopsis pilosula polysaccharides: Structural features and bioactivities diversity. Trends Food Sci Technol. (2020) 103:1–11. doi: 10.1016/j.tifs.2020.06.012

[64] Li F Yang F. Research progress on the extraction, separation, chemical composition, and pharmacological effects of codonopsis polysaccharides. Chin J Tradit Chin Med. (2023) 41:42–9. doi: 10.13193/j.issn.1673-7717.2023.04.008

[65] Lu J Yuan Q Yang Z Huang C Pei Ling T Li Z. Research progress on the extraction, separation, purification, structural characterization, and biological activity of polysaccharides from sugarcane leaves and bagasse. Food Industry Sci Technol. (2024), 1–20. doi: 10.13386/j.issn1002-0306.2024090025

[66] Zhou L Guo S-Y Cai M-Y Li L. Determination of the molecular weight of folded polysaccharides in aqueous solution by viscosity method. J Funct Polym. (2005) 04):692–5. doi: 10.14133/j.cnki.1008-9357.2005.04.029

[67] Wu Y-L Wang Y-L Yang L. Application of SEC-RI-MALLS technology in the analysis of molecular weight and molecular weight distribution of shiitake mushroom polysaccharides. J Pharm Anal. (2011) 31:2256–9. doi: 10.16155/j.0254-1793.2011.12.017

[68] Cao L Du C Zhai X Li J Meng J Shao Y. Codonopsis pilosula polysaccharide improved spleen deficiency in mice by modulating gut microbiota and energy related metabolisms. Front Pharmacol. (2022) 13:862763. doi: 10.3389/fphar.2022.862763, PMID: 35559259 PMC9086242

[69] Liu W Lv X Huang W Yao W Gao X. Characterization and hypoglycemic effect of a neutral polysaccharide extracted from the residue of Codonopsis Pilosula. Carbohydr Polym. (2018) 197:215–26. doi: 10.1016/j.carbpol.2018.05.067, PMID: 30007607

[70] Su Q Huo J Wang Y Zhou Y Luo D Hou J. The obesity amelioration effect in high-fat-diet fed mice of a homogeneous polysaccharide from Codonopsis pilosula. Molecules. (2022) 27. doi: 10.3390/molecules27165348, PMID: 36014584 PMC9415953

[71] Huang L Zhang H Xia W Yao N Xu R He Y. Structural characteristics, biological activities and market applications of Rehmannia Radix polysaccharides: A review. Int J Biol Macromol. (2024) 282. doi: 10.1016/j.ijbiomac.2024.136554, PMID: 39423982

[72] Zou YF Chen XF Malterud KE Rise F Barsett H Inngjerdingen KT. Structural features and complement fixing activity of polysaccharides from Codonopsis pilosula Nannf. var. modesta L.T.Shen roots. Carbohydr Polym. (2014) 113:420–9. doi: 10.1016/j.carbpol.2014.07.036, PMID: 25256503

[73] Zou Y Yan H Li C Wen F Jize X Zhang C. A Pectic Polysaccharide from Codonopsis pilosula Alleviates Inflammatory Response and Oxidative Stress of Aging Mice via Modulating Intestinal Microbiota-Related Gut-Liver Axis. Antioxid (Basel). (2023) 12. doi: 10.3390/antiox12091781, PMID: 37760084 PMC10525188

[74] Zhang L Yang Y. Determination of monosaccharide composition and content of codonopsis polysaccharides by high-performance liquid chromatography. China Food Additives. (2021) 32:163–9. doi: 10.19804/j.issn1006-2513.2021.12.022

[75] Jing Y Li A Liu Z Yang P Wei J Chen X. Absorption of Codonopsis pilosula saponins by coexisting polysaccharides alleviates gut microbial dysbiosis with dextran sulfate sodium-induced colitis in model mice. BioMed Res Int. (2018) 2018:1781036. doi: 10.1155/2018/1781036, PMID: 30211217 PMC6120299

[76] Ma K Yi X Yang ST Zhu H Liu TY Jia SS. Isolation, purification, and structural characterization of polysaccharides from Codonopsis pilosula and its therapeutic effects on non-alcoholic fatty liver disease *in vitro* and in *vivo* . Int J Biol Macromol. (2024) 265:130988. doi: 10.1016/j.ijbiomac.2024.130988, PMID: 38518942

[77] Meng X Kuang H Wang Q Zhang H Wang D Kang T. A polysaccharide from Codonopsis pilosula roots attenuates carbon tetrachloride-induced liver fibrosis via modulation of TLR4/NF-kappaB and TGF-beta1/Smad3 signaling pathway. Int Immunopharmacol. (2023) 119:110180. doi: 10.1016/j.intimp.2023.110180, PMID: 37068337

[78] Ming K He M Su L Du H Wang D Wu Y. The inhibitory effect of phosphorylated Codonopsis pilosula polysaccharide on autophagosomes formation contributes to the inhibition of duck hepatitis A virus replication. Poult Sci. (2020) 99:2146–56. doi: 10.1016/j.psj.2019.11.060, PMID: 32241500 PMC7587719

[79] Li LX Chen MS Zhang ZY Paulsen BS Rise F Huang C. Structural features and antioxidant activities of polysaccharides from different parts of Codonopsis pilosula var. modesta (Nannf.) L. T. Shen. Front Pharmacol. (2022) 13:937581. doi: 10.3389/fphar.2022.937581, PMID: 36091763 PMC9449496

[80] Zou YF Zhang YY Fu YP Inngjerdingen KT Paulsen BS Feng B. A polysaccharide isolated from Codonopsis pilosula with immunomodulation effects both *in vitro* and *in vivo* . Molecules. (2019) 24. doi: 10.3390/molecules24203632, PMID: 31600890 PMC6832355

[81] Ji HY Yu J Jiao JS Dong XD Yu SS Liu AJ. Ultrasonic-assisted extraction of Codonopsis pilosula glucofructan: optimization, structure, and immunoregulatory activity. Nutrients. (2022) 14. doi: 10.3390/nu14050927, PMID: 35267905 PMC8912531

[82] Zou YF Zhang YY Paulsen BS Rise F Chen ZL Jia RY. Structural features of pectic polysaccharides from stems of two species of Radix Codonopsis and their antioxidant activities. Int J Biol Macromol. (2020) :159:704–713. doi: 10.1016/j.ijbiomac.2020.05.083, PMID: 32422266

[83] Yang C Gou Y Chen J An J Chen W Hu F. Structural characterization and antitumor activity of a pectic polysaccharide from Codonopsis pilosula. Carbohydr Polym. (2013) 98:886–95. doi: 10.1016/j.carbpol.2013.06.079, PMID: 23987425

[84] Zhang Y-J Zhang L-X Yang J-F Liang Z-Y. Structure analysis of water-soluble polysaccharide CPPS3 isolated from Codonopsis pilosula. Fitoterapia. (2010) 81:157–61. doi: 10.1016/j.fitote.2009.08.011, PMID: 19686809

[85] Yongxu S Jicheng L. Structural characterization of a water-soluble polysaccharide from the Roots of Codonopsis pilosula and its immunity activity. Int J Biol Macromol. (2008) 43:279–82. doi: 10.1016/j.ijbiomac.2008.06.009, PMID: 18640151

[86] Locati M Curtale G Mantovani A. Diversity, mechanisms, and significance of macrophage plasticity. Annu Rev Pathol. (2020) 15:123–47. doi: 10.1146/annurev-pathmechdis-012418-012718, PMID: 31530089 PMC7176483

[87] Anderson NR Minutolo NG Gill S Klichinsky M. Macrophage-based approaches for cancer immunotherapy. Cancer Res. (2021) 81:1201–8. doi: 10.1158/0008-5472.Can-20-2990, PMID: 33203697

[88] Shi B Hu J Li P Xu K. Immunomodulatory effects of Codonopsis pilosula polysaccharides on ana-1 macrophages and mice. Biotechnol Bull. (2019) 35:114–8. doi: 10.13560/j.cnki.biotech.bull.1985.2018-0889

[89] Kennedy R Celis E. Multiple roles for CD4+ T cells in anti-tumor immune responses. Immunol Rev. (2008) 222:129–44. doi: 10.1111/j.1600-065X.2008.00616.x, PMID: 18363998

[90] Ashton-Rickardta PG Opferman JT. Memory T lymphocytes. Cell Mol Life Sci. (1999) 56:69–77. doi: 10.1007/s000180050007, PMID: 11213263 PMC11147043

[91] Liu K Xie S Li T Wang B Zhang H Zhu G. Study on the effect of Codonopsis pilosula polysaccharides on enhancing immune activity in chickens. Heilongjiang Anim Husbandry Vet Med. (2021) 13):116–9. doi: 10.13881/j.cnki.hljxmsy.2020.09.0215

[92] Wang J Zhang H Sun J Xu X. Effect of Polysaccharides from Codonopsis pilosula var. albostriata on Intestinal Mucosal Immune Function in Mice. Western J Tradit Chin Med. (2024) 37:10–4.

[93] Mauri C Bosma A. Immune regulatory function of B cells. Annu Rev Immunol. (2012) 30:221–41. doi: 10.1146/annurev-immunol-020711-074934, PMID: 22224776

[94] Carroll MC. The complement system in B cell regulation. Mol Immunol. (2004) 41:141–6. doi: 10.1016/j.molimm.2004.03.017, PMID: 15159059

[95] Fan Y Long Y Gong Y Gao X Zheng G Ji H. Systemic immunomodulatory effects of Codonopsis pilosula glucofructan on S180 solid-tumor-bearing mice. Int J Mol Sci. (2023) 24. doi: 10.3390/ijms242115598, PMID: 37958581 PMC10649278

[96] Wang D Liu Y Zhao W. The adjuvant effects on vaccine and the immunomodulatory mechanisms of polysaccharides from traditional chinese medicine. Front Mol Biosci. (2021) 8:655570. doi: 10.3389/fmolb.2021.655570, PMID: 33869288 PMC8047473

[97] Fabian KP Hodge JW. The emerging role of off-the-shelf engineered natural killer cells in targeted cancer immunotherapy. Mol Ther Oncol. (2021) 23:266–76. doi: 10.1016/j.omto.2021.10.001, PMID: 34761106 PMC8560822

[98] Wu SY Fu T Jiang YZ Shao ZM. Natural killer cells in cancer biology and therapy. Mol Cancer. (2020) 19:120. doi: 10.1186/s12943-020-01238-x, PMID: 32762681 PMC7409673

[99] Nicholson LB. The immune system. Essays Biochem. (2016) 60:275–301. doi: 10.1042/ebc20160017, PMID: 27784777 PMC5091071

[100] Borish LC Steinke JW. 2. Cytokines and chemokines. J Allergy Clin Immunol. (2003) 111:S460–75. doi: 10.1067/mai.2003.108, PMID: 12592293

[101] Fu YP Feng B Zhu ZK Feng X Chen SF Li LX. The polysaccharides from Codonopsis pilosula modulates the immunity and intestinal microbiota of cyclophosphamide-treated immunosuppressed mice. Molecules. (2018) 23. doi: 10.3390/molecules23071801, PMID: 30037030 PMC6100181

[102] Tang H Gu Y Jiang L Zheng G Pan Z Jiang X. The role of immune cells and associated immunological factors in the immune response to spinal cord injury. Front Immunol. (2022) 13:1070540. doi: 10.3389/fimmu.2022.1070540, PMID: 36685599 PMC9849245

[103] Parkin J Cohen B. An overview of the immune system. Lancet. (2001) 357:1777–89. doi: 10.1016/s0140-6736(00)04904-7, PMID: 11403834

[104] Li P Hu J Shi B. Analysis of Anti-complement Activity of Polysaccharides from Codonopsis pilosula var. modesta. Life Sci Res. (2018) 22:136–42. doi: 10.16605/j.cnki.1007-7847.2018.02.007

[105] Fu J Chang C Ren J Cheng J Gao Z Meng Y. The structure characterization of polysaccharides from Codonopsis pilosula and the structure-activity relationship with immune-regulation on THP-1 cells. Chem Biodivers. (2024) 25:e202401167. doi: 10.1002/cbdv.202401167, PMID: 39450708

[106] Lin X Fang C Ke W. The effect of Codonopsis pilosula polysaccharides on T-cell immune disorder and airway inflammation in rats with chronic obstructive pulmonary disease by regulating NF-κB signaling pathway. Tianjin Univ Tradit Chin Med. (2021) 38:788–93.

[107] Shi F Wang D Cao J Yu S. Immunomodulatory effect of bicontinuous Codonopsis pilosula polysaccharide nanoemulsion on immunosuppressed mice. Chin J Anim Nutr. (2020) 32:5925–31.

[108] Liu K Xie S. Study on the antioxidant activity of Codonopsis pilosula polysaccharide and its effect on serum immune and antioxidant functions of laying hens. Feed Res. (2024) 47:56–60. doi: 10.13557/j.cnki.issn1002-2813.2024.06.011

[109] Li C Yin L Zhu D He Y Li M Ding X. The effect of Codonopsis pilosula polysaccharides on intestinal mucosal immune function in piglets. Jiangsu J Agric Sci. (2018) 34:347–55.

[110] Andreas B-D Erik PS H NP Terje E Eirik MT. Dual inhibition of complement and Toll-like receptors as a novel approach to treat inflammatory diseases-C3 or C5 emerge together with CD14 as promising targets. J leukocyte Biol. (2017) 101:193–204., PMID: 27581539 10.1189/jlb.3VMR0316-132R PMC5166441

[111] Lans S Bardoel BW Ruyken M Haas C Baijens S Muts RM. Agnostic B cell selection approach identifies antibodies against K. pneumoniae that synergistically drive complement activation. Nat Commun. (2024) 15:8100–0. doi: 10.1038/s41467-024-52372-9, PMID: 39285158 PMC11405761

[112] Sui F Chen J Ma X Li H Liu X. Effect of Codonopsis pilosula polysaccharide on growth performance, serum immune indexes and antioxidant indexes of weaned piglets. Feed Industry. (2024) 45:38–42. doi: 10.13302/j.cnki.fi.2024.07.007

[113] Zhu G Hu Y Cao K Wang B. The effects of sulfated tremella polysaccharides and codonopsis polysaccharides on T lymphocyte proliferation and interleukin-2 mRNA expression levels in chickens. J Anim Nutr. (2017) 29:2535–40.

[114] Gong Z Zhang S Gu B Cao J Mao W Yao Y. Codonopsis pilosula polysaccharides attenuate Escherichia coli-induced acute lung injury in mice. Food Funct. (2022) 13:7999–8011. doi: 10.1039/d2fo01221a, PMID: 35818994

[115] Nie C Lan J Guo H Ouyang Q Zhao Y Wang P. Codonopsis pilosula polysaccharides (CPP) intervention alleviates sterigmatocystin (STC)-induced liver injury and gut microbiota dysbiosis. Int J Biol Macromol. (2024) 275:133190. doi: 10.1016/j.ijbiomac.2024.133190, PMID: 38897503

[116] Li Z Zhu L Zhang H Yang J Zhao J Du D. Protective effect of a polysaccharide from stem of Codonopsis pilosula against renal ischemia/reperfusion injury in rats. Carbohydr Polym. (2012) 90:1739–43. doi: 10.1016/j.carbpol.2012.07.062, PMID: 22944441

[117] Qian X Zhang X Ma R Du X. Microwave-assisted extraction and content analysis of polysaccharides from Codonopsis pilosula produced in Guizhou. Yunnan Chem Industry. (2022) 49:40–2.

[118] Liu Y Wang Y Mao D Chang K. Study on the isolation, purification and structural characterization of polysaccharides from codonopsis lanceolata. Food Machinery. (2023) 39:162–168+240. doi: 10.13652/j.spjx.1003.5788.2023.80573

[119] Su Y Xi J Shi Q Huang Y Zhao K Yang F. Research progress on Codonopsis pilosula (a medicinal and edible homologous Chinese herb). Chin Herbal Medicines. (2023) 54:2607–17.

[120] Wang Y Xu H Gao J Wen J Zhang Q Peng X. Research progress on preparation technology, structural characteristics, biological activities and pharmacokinetics of Codonopsis pilosula polysaccharides. Chin Herbal Medicines. (2025) 56:2617–28.

[121] Liu Z Yao X Xiao S Chen X Wu Q Yu L. Isolation, purification and structural analysis of a water-soluble polysaccharide from Codonopsis pilosula. Genomics Appl Biol. (2016) 35:1294–9. doi: 10.13417/j.gab.035.001294

