# Supplementary figures and images for: Preparation, structural characteristics and immune regulatory effects of Codonopsis pilosula polysaccharides: a review
Source: Front Immunol. 2025 Sep 22;16:1641928. doi: 10.3389/fimmu.2025.1641928 (PMC12497736; doi:10.3389/fimmu.2025.1641928)

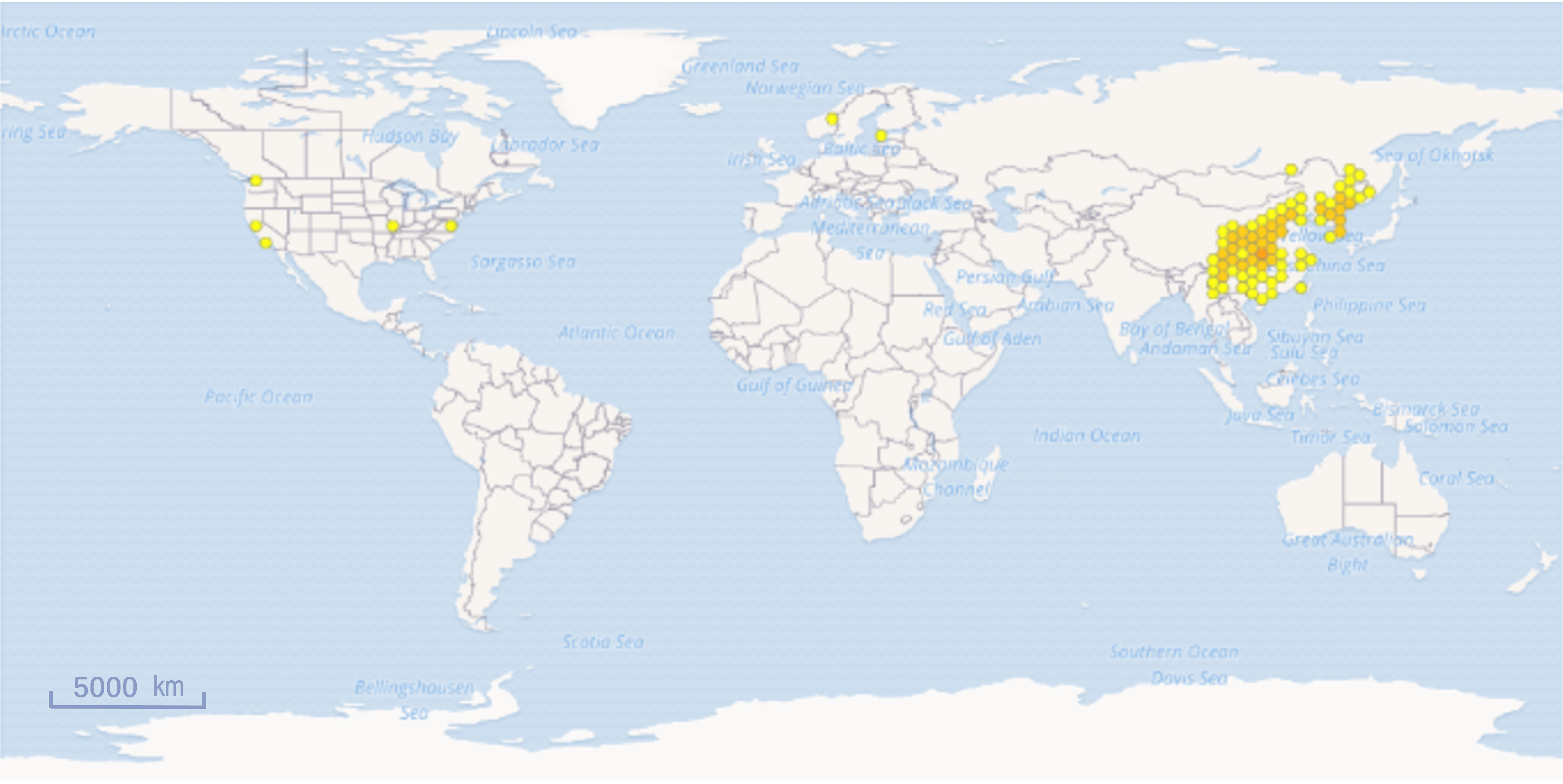

Supplement: Supplementary file 1 [file Image1.tif]
