# Supplementary material for: Preparation, structural characteristics and immune regulatory effects of Codonopsis pilosula polysaccharides: a review
Source: Front Immunol. 2025 Sep 22;16:1641928. doi: 10.3389/fimmu.2025.1641928 (PMC12497736; doi:10.3389/fimmu.2025.1641928)
Supplement: Supplementary file 3 [file Table1.docx]

**Supplementary Table 1**

Separation and purification of CPPs.

| Name | Extraction Method | Purification Methods | Ref. |
| --- | --- | --- | --- |
| CPP1a | HWE (90°C, 2h, 10ml/g, 3 cycles) | EPM, DEAE-52 cellulose | ([1](#_ENREF_1)) |
| RCNP | HWE (2h, 3 cycles) | EPM, DEAE-650M, Superdex G-200 | ([2](#_ENREF_2)) |
| RCAP-1 | HWE (2h, 3 cycles) | EPM, DEAE-650M, Superdex G-200 | ([2](#_ENREF_2)) |
| RCAP-2 | HWE (2h, 3 cycles) | EPM, DEAE-650M, Superdex G-200 | ([2](#_ENREF_2)) |
| CPPs-D1N1 | HWE (0.5h, 6ml/g) | EPM, DEAE-cellulose, Sephacryl S-400 | ([3](#_ENREF_3)) |
| CPP1-2-1 | HWE (1.5h) | EPM, DEAE Sepharose, Sephadex G-100/150 | ([4](#_ENREF_4)) |
| CPSP-1 | HWE (100°C, 2h, 2 cycles) | EPM, DEAE-Sepharose, Superdex 200 | ([5](#_ENREF_5)) |
| CTSP-1 | HWE (100°C, 2h, 2 cycles) | EPM, DEAE-Sepharose, Superdex 200 | ([5](#_ENREF_5)) |
| CPPF | HWE (100°C, 2.5h, 40ml/g, 2 cycles) | EPM, DEAE-Sepharose | ([6](#_ENREF_6)) |
| CPP | HWE | EPM, DEAE-52, Sephadex | ([7](#_ENREF_7)) |
| S-CPPA1 | HWE | EPM, DEAE-cellulose, Sepharose CL-6B | ([8](#_ENREF_8)) |
| 50WCP-II-I | HWE (50°C) | EPM, ANX Sepharose, Superdex 200 | ([9](#_ENREF_9)) |
| 100WCP-II-I | HWE (100°C) | EPM, ANX Sepharose, Superdex 200 | ([9](#_ENREF_9)) |
| CPP-1 | HWE (100°C, 1h, 2 cycles) | EPM, DEAE-52 | ([10](#_ENREF_10)) |
| CPPs | UAE | EPM, Sephadex G-25 | ([11](#_ENREF_11)) |
| CPP | HWE (100°C, 3 cycles) | Not specified | ([12](#_ENREF_12)) |
| CPPS-I | HWE (2h, 20ml/g, 3 cycles) | Macroporous resin, Ultrafiltration | ([13](#_ENREF_13)) |
| CPPS-II | HWE (2h, 20ml/g, 3 cycles) | Macroporous resin, Ultrafiltration | ([13](#_ENREF_13)) |
| CPPS-III | HWE (2h, 20ml/g, 3 cycles) | Macroporous resin, Ultrafiltration | ([13](#_ENREF_13)) |
| CLRP-1 | HWE (100°C, 2h, 40ml/g, 2 cycles) | EPM, DEAE-Sepharose, Superdex 200 | ([14](#_ENREF_14)) |
| CPP-A-1 | HWE (85°C, 2.5h, 10ml/g, 3 cycles) | EPM, DEAE Sepharose, Sephadex G-200 | ([15](#_ENREF_15)) |
| CPPS3 | HWE (100°C, 2h, 5ml/g, 3 cycles) | Ultrafiltration, Sephadex G-75 | ([16](#_ENREF_16)) |
| CPP | HWE (75°C, 3h, 10ml/g, 4 cycles) | EPM, DEAE-Sepharose CL-6B, Sephadex G-75/25 | ([17](#_ENREF_17)) |

**References**

1. Bai R, Li W, Li Y, Ma M, Wang Y, Zhang J, Hu F. Cytotoxicity of two water-soluble polysaccharides from Codonopsis pilosula Nannf. var. modesta (Nannf.) L.T.Shen against human hepatocellular carcinoma HepG2 cells and its mechanism. Int J Biol Macromol. 2018 Dec;120(Pt B):1544-1550. Epub 2018/09/25. doi:10.1016/j.ijbiomac.2018.09.123. Cited in: Pubmed; PMID 30248423.

2. Sun QL, Li YX, Cui YS, Jiang SL, Dong CX, Du J. Structural characterization of three polysaccharides from the roots of Codonopsis pilosula and their immunomodulatory effects on RAW264.7 macrophages. Int J Biol Macromol. 2019 Jun 1;130:556-563. Epub 2019/03/05. doi:10.1016/j.ijbiomac.2019.02.165. Cited in: Pubmed; PMID 30831168.

3. Rong X, Shu Q. Modulating butyric acid-producing bacterial community abundance and structure in the intestine of immunocompromised mice with neutral polysaccharides extracted from Codonopsis pilosula. Int J Biol Macromol. 2024 Oct;278(Pt 3):134959. Epub 2024/08/24. doi:10.1016/j.ijbiomac.2024.134959. Cited in: Pubmed; PMID 39179083.

4. Meng Y, Xu Y, Chang C, Qiu Z, Hu J, Wu Y, Zhang B, Zheng G. Extraction, characterization and anti-inflammatory activities of an inulin-type fructan from Codonopsis pilosula. Int J Biol Macromol. 2020 Nov 15;163:1677-1686. Epub 2020/09/27. doi:10.1016/j.ijbiomac.2020.09.117. Cited in: Pubmed; PMID 32979437.

5. Zou YF, Zhang YY, Paulsen BS, Rise F, Chen ZL, Jia RY, Li LX, Song X, Feng B, Tang HQ, Huang C, Yin ZQ. Structural features of pectic polysaccharides from stems of two species of Radix Codonopsis and their antioxidant activities. Int J Biol Macromol. 2020 Sep 15;159:704-713. Epub 2020/05/19. doi:10.1016/j.ijbiomac.2020.05.083. Cited in: Pubmed; PMID 32422266.

6. Fu YP, Li LX, Zhang BZ, Paulsen BS, Yin ZQ, Huang C, Feng B, Chen XF, Jia RR, Song X, Ni XQ, Jing B, Wu FM, Zou YF. Characterization and prebiotic activity in vitro of inulin-type fructan from Codonopsis pilosula roots. Carbohydr Polym. 2018 Aug 1;193:212-220. Epub 2018/05/19. doi:10.1016/j.carbpol.2018.03.065. Cited in: Pubmed; PMID 29773375.

7. Feng G, Zhang XF. Production of a codonopsis polysaccharide iron complex and evaluation of its properties. Int J Biol Macromol. 2020 Nov 1;162:1227-1240. Epub 2020/07/03. doi:10.1016/j.ijbiomac.2020.06.210. Cited in: Pubmed; PMID 32615228.

8. Li Z, Zhu L, Zhang H, Yang J, Zhao J, Du D, Meng J, Yang F, Zhao Y, Sun J. Protective effect of a polysaccharide from stem of Codonopsis pilosula against renal ischemia/reperfusion injury in rats. Carbohydr Polym. 2012 Nov 6;90(4):1739-43. Epub 2012/09/05. doi:10.1016/j.carbpol.2012.07.062. Cited in: Pubmed; PMID 22944441.

9. Zou YF, Chen XF, Malterud KE, Rise F, Barsett H, Inngjerdingen KT, Michaelsen TE, Paulsen BS. Structural features and complement fixing activity of polysaccharides from Codonopsis pilosula Nannf. var. modesta L.T.Shen roots. Carbohydr Polym. 2014 Nov 26;113:420-9. Epub 2014/09/27. doi:10.1016/j.carbpol.2014.07.036. Cited in: Pubmed; PMID 25256503.

10. Ma K, Yi X, Yang ST, Zhu H, Liu TY, Jia SS, Fan JH, Hu DJ, Lv GP, Huang H. Isolation, purification, and structural characterization of polysaccharides from Codonopsis pilosula and its therapeutic effects on non-alcoholic fatty liver disease in vitro and in vivo. Int J Biol Macromol. 2024 Apr;265(Pt 2):130988. Epub 2024/03/23. doi:10.1016/j.ijbiomac.2024.130988. Cited in: Pubmed; PMID 38518942.

11. Ji HY, Yu J, Jiao JS, Dong XD, Yu SS, Liu AJ. Ultrasonic-Assisted Extraction of Codonopsis pilosula Glucofructan: Optimization, Structure, and Immunoregulatory Activity. Nutrients. 2022 Feb 22;14(5). Epub 2022/03/11. doi:10.3390/nu14050927. Cited in: Pubmed; PMID 35267905.

12. Cao L, Du C, Zhai X, Li J, Meng J, Shao Y, Gao J. Codonopsis pilosula Polysaccharide Improved Spleen Deficiency in Mice by Modulating Gut Microbiota and Energy Related Metabolisms. Front Pharmacol. 2022;13:862763. Epub 2022/05/14. doi:10.3389/fphar.2022.862763. Cited in: Pubmed; PMID 35559259.

13. Li N, Xiong YX, Ye F, Jin B, Wu JJ, Han MM, Liu T, Fan YK, Li CY, Liu JS, Zhang YH, Sun GB, Zhang Y, Dong ZQ. Isolation, Purification, and Structural Characterization of Polysaccharides from Codonopsis pilosula and Their Anti-Tumor Bioactivity by Immunomodulation. Pharmaceuticals (Basel). 2023 Jun 19;16(6). Epub 2023/06/28. doi:10.3390/ph16060895. Cited in: Pubmed; PMID 37375842.

14. Li LX, Chen MS, Zhang ZY, Paulsen BS, Rise F, Huang C, Feng B, Chen XF, Jia RY, Ding CB, Feng SL, Li YP, Chen YL, Huang Z, Zhao XH, Yin ZQ, Zou YF. Structural features and antioxidant activities of polysaccharides from different parts of Codonopsis pilosula var. modesta (Nannf.) L. T. Shen. Front Pharmacol. 2022;13:937581. Epub 2022/09/13. doi:10.3389/fphar.2022.937581. Cited in: Pubmed; PMID 36091763.

15. Meng X, Kuang H, Wang Q, Zhang H, Wang D, Kang T. A polysaccharide from Codonopsis pilosula roots attenuates carbon tetrachloride-induced liver fibrosis via modulation of TLR4/NF-kappaB and TGF-beta1/Smad3 signaling pathway. Int Immunopharmacol. 2023 Jun;119:110180. Epub 2023/04/18. doi:10.1016/j.intimp.2023.110180. Cited in: Pubmed; PMID 37068337.

16. Zhang Y-j, Zhang L-x, Yang J-f, Liang Z-y. Structure analysis of water-soluble polysaccharide CPPS3 isolated from Codonopsis pilosula. Fitoterapia. 2010;81(3):157-161. doi:10.1016/j.fitote.2009.08.011.

17. Yongxu S, Jicheng L. Structural characterization of a water-soluble polysaccharide from the Roots of Codonopsis pilosula and its immunity activity. International Journal of Biological Macromolecules. 2008;43(3):279-282. doi:10.1016/j.ijbiomac.2008.06.009.
